# Proton‐Coupled Electron Transfer Deoxygenation of Pyridine N‐Oxide: A Mechanistic Study

**DOI:** 10.1002/cphc.202500292

**Published:** 2025-09-21

**Authors:** Céline Naddour, Gabriel Durin, Sylvie Chardon‐Noblat, Cyrille Costentin

**Affiliations:** ^1^ Université Grenoble Alpes, DCM, CNRS 38000 Grenoble France

**Keywords:** concerted dissociative electron transfer, N—O bond cleavages, proton‐coupled electron transfer, pyridine N‐oxides, reductive deoxygenation

## Abstract

Electrochemical reductive deoxygenation of pyridine N‐oxide is investigated with particular focus on the role of proton‐coupled electron transfers. A detailed analysis of cyclic voltammograms reveals that the initial electron transfer is followed by protonation of the pyridine N‐oxide anion radical. Kinetic analysis reveals an unusual fifth‐order dependence on the concentration of the proton donor (either water or ethanol), suggesting the involvement of a proton donor cluster in the protonation step. The resulting neutral radical represents a key bottleneck in the reaction pathway, as it can proceed via either a parent‐child coupling reaction or N—O bond cleavage, the latter leading to the formation of pyridine. This competition between reaction pathways allows extraction of both the rate constant for the protonation of the N‐oxide radical anion and kinetic information related to the reductive N—O bond cleavage. The reductive cleavage of the protonated N‐oxide radical may proceed via two possible mechanisms: 1) homolytic bond cleavage followed by reduction of the hydroxyl radical, or 2) a concerted dissociative electron transfer. The observed hydrogen‐bonding effects, combined with the higher driving force for the concerted pathway, support the latter mechanism, where stabilization of the departing hydroxide ion facilitates the electron transfer.

## Graphical abstract

This study explores the electrochemical reductive deoxygenation of pyridine N‐oxide, highlighting proton‐coupled electron transfers. A first stepwise electron–proton transfer leads to the protonated pyridine N‐oxide radical. Kinetic data support a concerted dissociative electron transfer mechanism over homolytic cleavage for N—O bond in the subsequent reductive sequence.

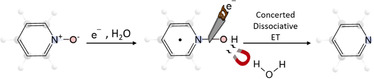

## 1 Introduction

The reductive deoxygenation of pyridine N‐oxides has been extensively studied,^[^ ^1^, ^2^ ^]^ not only due to the role of heterocyclic N‐oxides in natural processes^[^ ^3^, ^4^ ^]^ but also because of their widespread use as directing groups in the functionalization of oxygenated heterocyclic substrates.^[^ ^5^ ^]^ As a result, selective deoxygenation represents a fundamental transformation in organic synthesis. Traditionally, this reaction has required harsh conditions; however, over the past decade, several milder approaches have emerged, including photoredox catalysis,^[^ ^6^, ^7^ ^]^ photocatalysis,^[^ ^8^ ^]^ catalyst‐free photoreduction,^[^ ^9^, ^10^ ^]^ or electrochemical methods.^[^ ^11^, ^12^ ^]^ In all cases, proton transfers are proposed to play a crucial role with various mechanisms when investigated; for example, initial protonation of the N‐oxide,^[^ ^6^ ^]^ or proton‐coupled electron transfer (PCET) from a photoacid.^[^ ^9^ ^]^ The coupling of electron transfer (ET) and proton transfer has been investigated from the early days of mechanistic studies of heterocyclic N‐oxide reductive deoxygenation in particular in water.^[^ ^2^ ^]^ However, the actual mechanism in organic solvents is not yet fully understood.^[^ ^2^, ^13^ ^]^ In this work, we investigate the direct reductive deoxygenation of pyridine N‐oxide at a glassy carbon electrode (GCE) in acetonitrile with particular emphasis on the role of water as a proton donor. Using classical tools of molecular electrochemistry, combined with density functional theory (DFT) calculations, we uncover the complexity of the reaction mechanism. Our findings suggest that the N—O bond cleavage proceeds via a stepwise sequence of ET followed by proton transfer where the protonation step exhibits an unusual fifth‐order dependence on the proton donor. This is followed by a concerted dissociative ET (DET), likely assisted by hydrogen bonding that stabilizes the departing hydroxide ion.

## 2 Results and Discussion

### 2.1 Electroreduction of Pyridine N‐Oxide Without Added Water

**Scheme 1:**
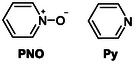
Pyridine N‐oxide (PNO) and pyridine (Py).

**Figure 1:**
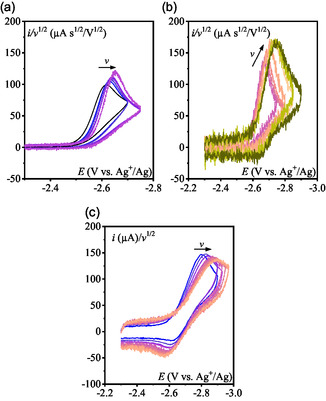
CVs in CH_3_CN + 0.1 M *n*‐Bu_4_NPF_6_ under Ar on a 3 mm‐diameter GCE a) PNO 1 mM at *ν* = 0.05, 0.07, 0.1, 0.2, 0.5 V s^−1^ (uncompensated resistance: 80 Ω). b) 0.7, 1, 2, 5, 7, 10 V s^−1^ (uncompensated resistance: 80 Ω). c) 15, 20, 25, 30, 35, 40, 45 V s^−1^ (uncompensated resistance: 180 Ω).

Cyclic voltammograms (CV) of pyridine N‐oxide (**Scheme** **1**) were recorded in acetonitrile containing 0.1 M *n*‐Bu_4_NPF_6_ under Ar. In these conditions, the residual water content has been determined by Karl‐Fischer titration to be 5 mM.^[^ ^14^ ^]^ Between 0.05 and 10 V s^−1^, the CV exhibits chemically irreversible behavior (**Figure** **1**a,b). However, at higher scan rates (*v* > 15 V s^−1^), partial reversibility is observed (Figure 1c). No additional reduction is detected up to −3 V vs. Ag^+^/Ag (electrolyte reduction; Figure S1, Supporting Information).

Normalization of the CVs by the square root of the scan rate (*v*) reveals that, starting from the highest scan rates, the cathodic peak current, $i_{p}/\sqrt{v}$, increases slightly as the scan rate decreases, followed by a substantial drop (Figure 1). At the highest scan rate (45 V s^−1^), the observed partial chemical reversibility suggests that the peak current should approach the value predicted by the Randles–Sevcik Equation (1) for a one‐electron reversible process

(1) $$i_{p}^{0}=0.446FSC^{0}\sqrt{DFv/RT}$$

However, the measured peak current is lower, primarily due to the effect of ohmic drop. From the capacitive charging current, the solution resistance between the working electrode (GCE) and the reference electrode was estimated to be 180 Ω and the capacitance of the electrode estimated to be 4–5 μF (Figure S2, Supporting Information). Based on these values, the peak current for a 1 mM monoelectronic reversible wave for PNO reduction at a 0.07 cm^2^ GCE was simulated accounting for the ohmic drop. Fitting the simulation to the experimental data at 45 V s^−1^ yielded a diffusion coefficient of *D* = 6 × 10^−5^ cm^2^ s^−1^ (Figure S3, Supporting Information). Additionally, the position of the cathodic peak allowed determination of the standard potential of the PNO/PNO^•−^ couple: *E* ^0^ = −2.675 V versus Ag^+^/Ag (Figure S3, Supporting Information). This value aligns well with a previously reported value at −30 °C in dimethylformamide^[^ ^13^ ^]^ as well as with the DFT‐calculated value of ≈−2.70 V versus Ag^+^/Ag (see Supporting Information for details).

**Figure 2:**
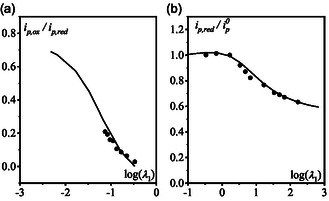
a) Ratio of anodic and cathodic current as function of $\log\lambda_{1}$, b) cathodic peak current normalized by the peak current of a reversible monoelectronic wave as function of $\log\lambda_{1}$. In both (a) and (b) *k*_1_ and *k*_2_ were adjusted so that the theoretical working curves (full lines) match with the experimental data (see text).

**Scheme 2:**
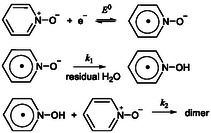
Mechanism of PNO electroreduction without added water.

The slight increase of the normalized cathodic current peak $i_{p}/\sqrt{v}$ observed when the scan rate is decreased from 45 to 10 V s^−1^ (Figure 1c) can be attributed to: 1) a decrease of the ohmic drop effect at lower scan rates, and 2) an inherent decrease in peak current as the system transitions from irreversible to reversible behavior. The decrease of $i_{p}/\sqrt{v}$ for scan rates below 10 V s^−1^ (Figure 1a,b) indicates that the effective stoichiometry of the wave is less than one. This stoichiometry can be estimated from the ratio of the observed peak current to $i_{p}^{0}$, which drops to 0.64 (**Figure** **2**b). Such a behavior is indicative of a “parent–child” type mechanism wherein the primary reaction step gives the electrogenerated intermediate which reacts with the initial substrate. This phenomenon has been extensively studied in systems involving self‐protonation reactions.^[^ ^15^ ^]^ In the present case, however, a self‐protonation mechanism is unlikely, as PNO lacks an acidic proton. Thus, alternative reaction pathway must be considered, pending further mechanistic insight. In conjunction with the stoichiometry, the degree of reversibility, defined as the ratio of the anodic to the cathodic peak currents, was analyzed as function of scan rate to evaluate whether the “parent–child” reaction is the actual chemical step following the initial ET. As shown in the Supporting Information (Section 4), no single rate constant can simultaneously account for the observed rate dependence of both the peak current ratio and the stoichiometry, effectively ruling out a “parent–child” mechanism as the primary pathway. Other simple mechanisms can also be ruled out (see Supporting Information, Section 5). A more plausible explanation involves a first‐order chemical step following the initial ET that generates the PNO^•−^ radical anion. One likely scenario is protonation of PNO^•−^ by residual water (5 mM), yielding the neutral PNOH^•^ radical. To account for the substoichiometric wave, a subsequent “parent–child” step must be invoked. We propose that PNOH^•^ reacts with a molecule of PNO to form a dimer, consistent with known behavior of protonated pyridine N‐oxides, which are reported to dimerize with PNO (**Scheme** **2**).^[^ ^16^ ^]^

A formal kinetic analysis of the mechanism, proposed in Scheme 2, reveals that the shape of the CV, specifically the degree of reversibility and apparent stoichiometry, is governed by two parameters $\lambda_{1}=k_{1}/\left(Fv/RT\right)$ and $\lambda_{2}=k_{2}C^{0}/\left(Fv/RT\right)$. These parameters define a 2D zone diagram (Figure S5, Supporting Information), where transitions between different kinetic regimes can be induced by varying the scan rate (Figure S6, Supporting Information). This approach enables unambiguous determination of both *k*_1_ and *k*_2_. In practice, the degree of reversibility is primarily sensitive to $\lambda_{1}=k_{1}/\left(Fv/RT\right)$. By fitting the experimental data to the corresponding theoretical working curve (Figure 2a), *k*_1_ was estimated to be 160 s^−1^. Subsequently, *k*_2_ was extracted by fitting the scan rate dependence of the apparent stoichiometry using the theoretical working curve (Figure 2b). We obtain *k*_2_ = 2 10^4^ M^−1^ s^−1^. The validity of this kinetic analysis was further confirmed through full simulations of the CVs across the entire range of scan rates, showing a good agreement between experimental and simulated data (Figure S7, Supporting Information).

A previous study of the chemical monoelectronic reduction of PNO in dry THF reported the formation of bipyridine via dimerization of the initially formed radical anion.^[^ ^17^ ^]^ However, such a pathway does not appear to occur under the present conditions at low scan rates based on the following experimental observations: 1) the apparent stoichiometry is less than one and 2) if bipyridine was formed, it would undergo further reversible reduction at the same potential, leading to an even higher stoichiometry. Interestingly, pyridine was also observed as a side product. In our case, exhaustive controlled potential electrolysis (CPE) at −2.72 V versus Ag^+^/Ag, on a carbon felt electrode of a 5.75 mM solution of PNO, led predominantly to the formation of Py with an overall consumption of two electrons per PNO molecule. We note that the shape of the current over time during the CPE (Figure S12, Supporting Information) indicates that most of the transformation is barely affected by mass transport of PNO, as evidenced by modification of the stirring rate of the solution during electrolysis (Figure S13, Supporting Information). This indicates that the carbon felt electrode acts as a sponge for PNO, facilitating electrochemical reduction within its porous structure, with rapid replenishment of substrate. Given these conditions, the mechanism inferred from CV analysis, on a planar GCE, may not accurately reflect the process occurring during bulk electrolysis. Nevertheless, the collective results highlight the need for a more detailed investigation into the role of water in steering the reaction pathway toward the formation of Py.

### 2.2 Electroreduction of Pyridine N‐Oxide in the Presence of a Proton Donor

**Figure 3:**
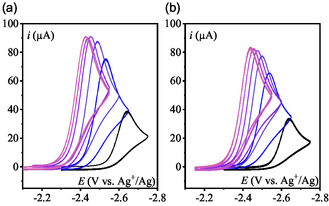
CVs in CH_3_CN + 0.1 M *n*‐Bu_4_NPF_6_ on a 3 mm‐diameter GCE at 0.1 V s^−1^. a) PNO 1.15 mM (black) + 0.1, 0.2, 0.3, 0.4, 0.5 M H_2_O b) PNO 1 mM (black) + 0.1, 0.2, 0.3, 0.4, 0.5 M D_2_O.

The addition of increasing amounts of water, from 100 to 500 mM, results in two key observations: 1) an anodic shift in the reduction wave of PNO and 2) an increase in current suggesting a higher overall stoichiometry of the process (**Figure** **3**a). Additionally, a second reduction wave emerges at more negative potentials, which also shifts as the water concentration increases. It corresponds to the reduction of Py in the presence of water (Figure S14, Supporting Information), confirming that Py is formed as a product on the timescale of CV.

The formation of Py during the electrochemical reduction of PNO in the presence of water was further confirmed by performing exhaustive CPE at −2.60 V versus Ag^+^/Ag on a carbon felt electrode using a 9.15 mM solution of PNO with 200 mM H_2_O. NMR analysis of the electrolyzed solution confirmed the formation 75% of Py after complete consumption of PNO with a faradaic yield of 55% (see Supporting Information for details; Figure S15, Supporting Information).

**Scheme 3:**
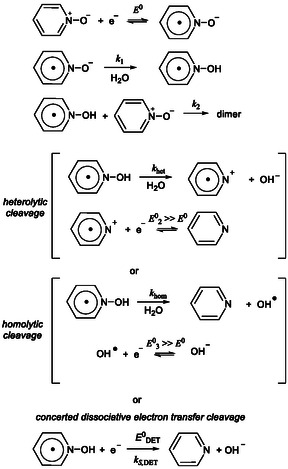
Mechanism of PNO electroreduction in the presence of water.

Taken together, these observations suggest that, in the presence of water, the reaction proceeds via a two‐electron reductive deoxygenation of PNO to form Py. Building on the mechanism discussed above (Scheme 2), we propose the pathways depicted in **Scheme** **3**. Our mechanistic proposal involves the rapid formation of the protonated radical intermediate, PNOH^•^, facilitated by the higher concentration of water (rate constant *k*_1_), followed by the cleavage of the N—O bond. This process not only bypasses the “parent–child” reaction observed in dry conditions, but also promotes a second ET, ultimately enabling the two‐electron reduction to Py. Note that we can rule out a stepwise pathway involving initial protonation of PNO followed by an ET, as is typical in acidic aqueous solutions. ^1a^ This scenario is unlikely here, as the *pK*_a_ of water in acetonitrile (*pK*_a_(H_2_O) ≈ 35^[^ ^18^ ^]^) is far too high to significantly protonate PNO, whose *pK*_a_ in acetonitrile has been estimated to be around 10.^[^ ^19^ ^]^ Additionally, a concerted proton–ET can be excluded, as it would result in significant broadening of the CV waves due to kinetic effects, which is not observed experimentally.^[^ ^20^ ^]^ Therefore, the most consistent mechanism involves an initial ET to PNO, followed by protonation of the resulting radical anion, that is, a stepwise electron–proton transfer sequence.

**Scheme 4:**
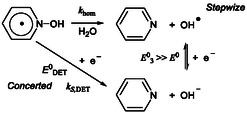
Reductive cleavage of PNOH^•^.

As illustrated in Scheme 3, we consider three possible mechanisms for the reductive cleavage of PNOH^•^. DFT calculations rule out the possibility of an initial reduction of PNOH^•^, as the formation of a stable PNOH^−^ species could not be computed without spontaneous dissociation. The first two proposed mechanisms are stepwise and involve initial bond cleavage of PNOH^•^, followed by reduction of the resulting radical. Such cleavage can occur either homolytically or heterolytically.^[^ ^21^ ^]^ However, DFT calculations show that while homolytic cleavage is thermodynamically favorable (Δ*G* ^0^ = −0.73 eV), heterolytic cleavage is uphill and would generate a highly energetic Py^•+^ species (Δ*G* ^0^ = 0.93 eV). As a result, we discard the heterolytic pathway. The third mechanism we propose is a concerted DET, wherein PNOH^•^ undergoes direct heterogeneous reduction concerted with N—O bond cleavage. This pathway is thermodynamically very favorable at the potentials at which PNOH^•^ is formed: DFT calculations estimate a standard potential of $E_{\text{DET}}^{0}\approx$ 1.05 V versus Ag^+^/Ag for this transformation, giving a driving force of ≈ 3.5 eV. Given this high thermodynamic driving force, such a reduction must be kinetically very slow under dry conditions, otherwise the CV would display a two‐electron process even in the absence of added water. Unfortunately, we are unable to simply distinguish between the two viable pathways: 1) stepwise homolytic cleavage of PNOH^•^ followed by reduction of the resulting OH^•^, and 2) a concerted dissociative reduction of PNOH^•^ (**Scheme** **4**). Indeed, both would lead to similar outcomes in terms of wave stoichiometry, while the position of the CV wave remains primarily determined by the rate constant for the protonation of PNO^•−^ to form PNOH^•^. Therefore, in our analysis of CVs in the presence of added water, we have considered both mechanistic possibilities as outlined in Scheme 4.

**Figure 4:**
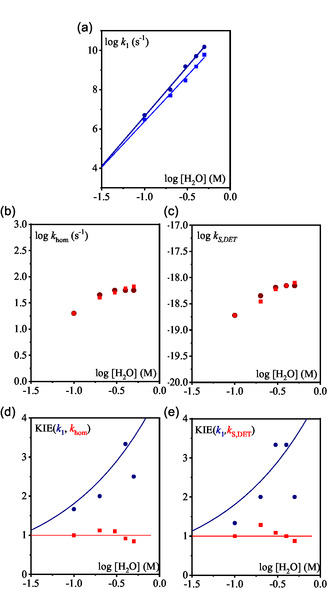
a) Rate constant *k*_1_ as function of added H_2_O (dark blue circle) and D_2_O (light blue square). Lines are linear fitting, b) rate constant *k*_hom_ as function of added H_2_O (dark red circle) and D_2_O (light red square). c) Rate constant *k*_S,DET_ as function of added H_2_O (dark red circle) and D_2_O (light red square). d) KIE on *k*_1_ (blue) and *k*_hom_ (red). e) KIE on *k*_1_ (blue) and *k*_S,DET_ (red).

Assuming that the rate constant *k*_2_ for the “parent–child” step is independent of the water content, the CVs can be analyzed in the framework of the mechanisms proposed in Scheme 3. This allows us to evaluate both *k*_1_ and *k*_hom_ (in the homolytic cleavage pathway) or *k*_1_ and *k*_S,DET_ (in the concerted DET pathway) as function of the water concentration. Focusing first on the homolytic cleavage scenario, the formal kinetic analysis of the mechanism (see Supporting Information) indicates that the system is governed by two parameters $\lambda_{\mathrm{hom}}/\lambda_{1}$ and 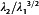 where $\lambda_{\mathrm{hom}}=k_{\mathrm{hom}}/\left(Fv/RT\right)$. Consequently, knowing *k*_2_, both *k*_1_ and *k*_hom_ can be extracted from the CV features (wave position and intensity) for any water concentration provided the system crosses different zones of the associated zone diagram. Experimentally, this condition is met: both the position and the stoichiometry of the CV wave change significantly with increasing water concentration at a fixed scan rate. Following this approach, both *k*_1_ and *k*_hom_ were adjusted and plotted as a function of the water concentration (**Figure** **4**a,b; see simulated CVs in Figure S8, Supporting Information).

The rate constant *k*_1_ shows a strong dependence on water concentration, with an apparent reaction order of 5. In contrast, the N—O bond cleavage rate constant *k*_hom_ also increases with water concentration but exhibits a lower reaction order, ≈1 at low water content and approaching zero at higher concentration. Repeating the same experiments using D_2_O instead of H_2_O yields similar qualitative trends (Figure 3b, S9, and S10, Supporting Information). However, quantitative analysis reveals that *k*_1_ values are significantly lower in D_2_O, while *k*_hom_ remains unchanged in both conditions (Figure 4a,b). This results in a substantial kinetic isotope effect (KIE) for *k*_1_, consistent with a protonation step. On the other hand, the absence of KIE for *k*_hom_, despite its sensitivity to water concentration, suggests that the N—O bond cleavage would involve hydrogen bonding with water rather than direct proton transfer (Figure 4c,e). It seems however surprising for a homolytic cleavage.

Turning to the alternative mechanism, namely a concerted dissociative ET for the reduction of PNOH^•^ to Py and OH^−^, the formal kinetic analysis of the mechanism (see Supporting Information) indicates that the system is also governed by two parameters 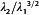 and $\Lambda =\frac{k_{\text{S,DET}}}{\sqrt{Dk_{1}}}$, assuming, for the sake of simplicity, a Butler–Volmer kinetic law for the concerted DET and also assuming that both the transfer coefficient *α* and the standard potential $E_{\text{DET}}^{0}$ are known. A more accurate kinetic law is the Marcus–Hush–Levich law^[^ ^22^, ^23^ ^]^ characterized by $E_{\text{DET}}^{0}$, the reorganization energy $\lambda_{\text{DET}}$ and $k_{\text{S,DET}}$. Using a standard potential of $E_{\text{DET}}^{0}=$ 1.05 V versus Ag^+^/Ag from DFT calculations and assuming $\lambda_{\text{DET}}\approx$4 eV (see discussion below for a posteriori justification of this value), we simulated CVs under the assumption that *k*_2_ is independent of the water content. This analysis allows the extraction of both *k*_1_ and $k_{\text{S,DET}}$ as function of water concentration (Figure 4a–c; simulated CVs in Figure S8, Supporting Information). Notably, since the wave position is primarily dictated by *k*_1_, its value remains consistent across both mechanistic scenarios. The same analysis was repeated with addition of D_2_O to determine the KIE on $k_{\text{S,DET}}$ (Figure 4e). As observed previously, *k*_1_ displays a reaction order of 5 with respect to water concentration and shows a pronounced KIE. In contrast, $k_{\text{S,DET}}$ exhibits no KIE, although it does depend on water concentration. These findings suggest that if the concerted DET pathway dominates for PNOH^•^ reductive cleavage, the reaction is facilitated by hydrogen‐bonding interactions with water (stabilizing the departing hydroxide ion), rather than by direct proton transfer.

**Figure 5:**
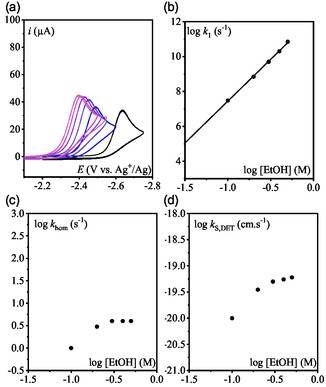
CVs in CH_3_CN + 0.1 M *n*‐Bu_4_NPF_6_ on a 3 mm‐diameter GCE at 0.1 V s^−1^. a) PNO 1 mM (black) + 0.1, 0.2, 0.3, 0.4, 0.5 M EtOH, b) rate constant *k*_1_ as function of added EtOH, and c) rate constant *k*_hom_ as function of added EtOH. d) Rate constant *k*_S,DET_ as function of added EtOH.

To further investigate the influence of proton donors, the reduction of PNO was examined using ethanol (EtOH) as an alternative proton source. Ethanol is slightly more acidic than water in acetonitrile (*pK*_a_(EtOH) ≈ 30^[^ ^24^, ^25^ ^]^ versus *pK*_a_(H_2_O) ≈ 35) but is a weaker hydrogen‐bond donor (HBD). Following the same methodology used with water, *k*_1_, *k*_hom_ and $k_{\text{S,DET}}$ were extracted from the CVs (**Figure** **5** and S11, Supporting Information). Notably, the value of *k*_1_ increases with EtOH concentration, displaying a reaction order of five, consistent with the behavior observed in the presence of water. However, the reaction proceeds more rapidly with EtOH, likely due to its stronger acidity providing a stronger driving force. In contrast, both the putative N—O bond cleavage rate constant *k*_hom_ and *k*_S,DET_ are slower with EtOH than with water, which aligns with EtOH's weaker hydrogen‐bond donating ability reflected in the α HBD parameter which quantifies a molecule's hydrogen‐bond donating strength (1.17 for water compared to 0.83 for EtOH^[^ ^26^ ^]^).

## 3 Discussion

The detailed mechanistic investigation of the direct reduction of PNO on a carbon electrode reveals that the N—O bond cleavage proceeds through a multistep process. Initially, PNO is electrochemically reduced to form its corresponding radical anion (PNO^•−^). This intermediate is then protonated, and the resulting protonated radical undergoes a reductive N—O bond cleavage either via a stepwise or a concerted mechanism. Cyclic voltammetry reveals a complex reaction stoichiometry, indicating the occurrence of a side “parent–child” reaction. This process is proposed to involve the reaction of the protonated radical (PNOH^•^) with the parent PNO.

**Scheme 5:**
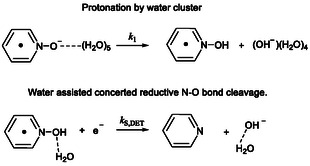
Proposed role of water in PNO reductive deoxygenation.

The first key chemical step preceding N—O bond cleavage is likely the protonation of PNO^•−^. We found that the apparent rate constant for this protonation exhibits a reaction order of five with respect to the proton donor, regardless of whether water or ethanol is used. This suggests that protonation occurs within a hydrogen‐bonded network or cluster of H_2_O molecules (**Scheme** **5**).^[^ ^27^, ^28^ ^]^ Furthermore, this observation implies that the resulting species may be more complex than a simple PNOH^•^ radical, potentially existing as a solvated or associated complex with the proton donor cluster, which may facilitate its subsequent reduction.

The fate of PNOH^•^ appears to be the kinetic bottleneck of the overall process, influencing the reaction stoichiometry toward values of 0.5 or 2. Importantly, it is this competition that enables the determination of the N—O bond cleavage rate constant via cyclic voltammetry, based on the evolution of reaction stoichiometry with increasing proton donor concentration. Two mechanistic pathways are considered for the reductive cleavage of PNOH^•^ (Scheme 4): 1) a stepwise mechanism involving homolytic N—O bond cleavage (HOM) followed by the reduction of the resulting hydroxyl radical or 2) a concerted DET. In both scenarios, the apparent rate constant shows a first‐order dependence on proton donor (that eventually becomes closer to zero‐order), displays no KIE and is faster with ethanol than with water as proton donor. These results strongly suggest that hydrogen‐bonding plays a role in facilitating N—O bond cleavage of PNOH^•^. In this context, the concerted DET appears more plausible, as the leaving hydroxide ion can be stabilized via hydrogen bonding to water or ethanol (Scheme 5). We also note that simulation of the CVs based on the concerted DET mechanism provide a better fit than those based on the stepwise homolytic pathway, especially on the reverse scan (Figures S8, S10, and S11, Supporting Information).

**Scheme 6:**
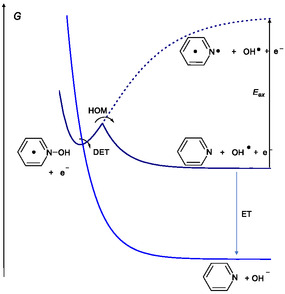
Sketch of free energy profiles.

Finally, a standard free energy diagram can be constructed to illustrate the possible mechanisms for the reductive cleavage of PNOH^•^, the key step to the formation of pyridine (**Scheme** **6**). Considering an applied potential of −2.50 V versus Ag^+^/Ag and a calculated standard potential $E_{\text{DET}}^{0}=$ 1.05 V versus Ag^+^/Ag, the driving force for the concerted DET is 3.55 eV. In contrast, DFT calculations estimate the driving force for the homolytic cleavage of PNOH^•^ to be 0.735 eV. This implies a driving force of 2.815 eV for the subsequent reduction of the resulting hydroxyl radical reduction equal. Consequently, the corresponding standard potential for hydroxyl radical reduction is calculated to be $E_{{\text{OH}}^{•}{\text{/OH}}^{-}}^{0}=$ 0.315 V versus Ag^+^/Ag. The ease to oxidize the leaving group, OH^−^, might be considered as the main favorable factor for the concerted DET.^[^ ^22^ ^]^

The very large driving force associated with the concerted DET renders it a viable mechanistic pathway, despite the high reorganization energy (≈ 4 eV) required, a value larger than typical values of substrates undergoing concerted DET.^[^ ^29^, ^30^ ^]^ This energy corresponds to the N—O bond dissociation energy but without electronic rearrangement of the two unpaired electron in Py, resulting in an excited state of Py (Scheme 6).^[^ ^21^, ^22^ ^]^ Considering a quadratic activation‐driving force relationship for the concerted DET,^[^ ^22^, ^29^ ^]^ the activation free energy is estimated to be only $\Delta G_{\text{DET}}^{\neq }=\frac{\lambda_{\text{DET}}}{4}{\left(1-\frac{F\left(E-E_{\text{DET}}^{0}\right)}{\lambda_{\text{DET}}}\right)}^{2}=0.016\text{eV}$, making this process the most favorable one in terms of activation barrier. Furthermore, the excitation energy associated with a $n\to \pi^{*}$ transition in Py is consistent with the reorganization energy used in this model, *λ*_DET_ = 4 eV. Indeed, we have $E_{\text{ex}}=\lambda_{\text{DET}}+0.735$ eV, corresponding to an absorption band at a wavelength of 262 nm. This aligns well with the experimental UV spectrum of pyridine, which displays a broad absorption band in the 220–310 nm region with a maximum near 260 nm.^[^ ^31^ ^]^ This serves as a posteriori validation of the value used for *λ*_DET_ in the analysis.

## 4 Conclusion

Through a comprehensive mechanistic study integrating electrochemical experimental data and DFT calculations, we elucidated the distinct PCET pathways involved in the deoxygenation of pyridine N‐oxide in acetonitrile in the presence of a proton donor. The initial PCET sequence proceeds via a stepwise mechanism, beginning with an ET followed by a proton transfer mediated through a hydrogen‐bonded proton donor cluster, ultimately forming a protonated PNOH^•^ radical. Cyclic voltammetry revealed a subsequent “parent–child” type transformation of this intermediate. The N—O bond cleavage occurs in a second PCET step involving the protonated radical, which experimental evidence indicates proceeds through a concerted DET, driven by a substantial thermodynamic driving force and solvation of the departing hydroxide ion.

## 5 Experimental Section

5.1

5.1.1

##### Chemicals

Unless otherwise stated, all the reagents were degassed and used as received. Acetonitrile (≥99.9%, gradient grade), pyridine N‐oxide (95%, nitrogen flushed), and EtOH (≥99.8%) were purchased from Fisher Chemical/Thermo‐scientific. *n*‐Bu_4_NPF_6_ (≥99%) was purchased from Sigma‐Aldrich chemicals. Pyridine (sds, ≥99.5%, anhydrous analytical grade) was distillated before use. H_2_O was obtained from a water purification system Elga (option Purelab, 18.2 MΩ cm). D_2_O (99.96% D) was purchased from Euriso‐top.

##### Cyclic Voltammetry

All cyclic voltammetry experiments were performed in an argon filled glovebox with a CHI 750 E bipostentiostat. Without specific indication in the text, when used, ohmic drop was compensated using the positive feedback compensation implemented in the instrument. A standard three‐electrode electrochemical cell was used. CV were obtained with a 3 mm‐diameter GCE. Prior to the acquisition, the working electrode was polished using 2 μm monocrystalline diamond paste PM (Presi) on a microcloth polishing pad. It was pretreated by cycling between upper and lower potential limits of the electrochemical window for the experiments in the electrolyte solution. The counter‐electrode was a platinum wire in *n*‐Bu_4_NPF_6_ (0.1 M) acetonitrile solution. The reference electrode was an Ag^+^/Ag electrode with a AgNO_3_ (10 mM), *n*‐Bu_4_NPF_6_ (0.1 M) solution in acetonitrile. Both were separated from the electrolytic working solution through an *n*‐Bu_4_NPF_6_ (0.1 M) acetonitrile solution bridge (glass frit). The potential of the reference electrode: *E* ^0^(ferrocene) = 0.09 V vs. Ag^+^/Ag was measured by running a CV of a solution of ferrocene (1 mM) and *n*‐Bu_4_NPF_6_ (0.1 M) in acetonitrile. Additions of proton source were made using a Hamilton microsyringe, directly in the cell solution, followed by stirring for homogenization. Dilution is taken into consideration.

##### CPE

All electrolysis experiments were performed in an argon filled glovebox using a Echem Lab xm solartron analytical potentiostat. The experiments were carried out in a conventional three‐electrode cell with a carbon felt working electrode, the volume of the CH_3_CN solution was 10 mL. The reference electrode and the counter electrode were as described above.

##### ^1^H NMR

NMR measurements were made using the Avance III 500 MHz Bruker. Data were analyzed on TopSpin 4.3.0. Mesitylene was used as internal standard for quantitative measurements. A solution of 5 mM mesitylene was prepared in CD_3_CN. Measurements were made by taking 150 μL of the electrochemical cell solution before and after electrolysis. 150 μL of the reference (mesitylene) solution were added to each NMR tubes. The CH_3_CN peak (1.94 ppm) was irradiated to minimize it using the PRESAT option. The aromatic region of the ^1^H NMR spectrum shows the peaks corresponding to pyridine N‐oxide (PNO; 7.71 ppm), pyridine (Py; 8.55 ppm) and mesitylene (6.78 ppm), it is the region of interest in this study. A calibration curve was established using solutions prepared in the same conditions as those for electrolysis (CH_3_CN + 0.1 M *n*‐Bu_4_NPF_6_), with known concentrations of Py and PNO, in the same range of order of magnitude of the reactants and products awaited (1–10 mM). The integrals of the peaks were used to calculate the concentrations of the compounds of interest (*x*) in the presence of the mesitylene (ref) using the following formula

$C_{x}=\frac{I_{x}}{I_{\text{ref}}}\frac{N_{\text{ref}}}{N_{x}}C_{\text{ref}}$ with *I*, *N,* and *C* being the integral area, the number of nuclei, and the concentration of the compound, respectively.

Taking into account the calibration curve, the yield can be calculated as follows $\text{Yield}(\%)=100\times \frac{C_{\text{Py,final}}}{C_{\text{PNO,initial}}}$

##### Karl‐Fischer Titration

Water titration in *n*‐Bu_4_NPF_6_ (0.1 M) acetonitrile solution was performed using the solvent standard method for water content determination with a Mettler Toledo C10S Compact Coulometric KF Titrator. The reagent for coulometric titration KF (anolyte solution) is Honeywell Fluka HYDRANAL‐Coulomat AG.

## Supporting Information

Additional data. CV simulations. DFT calculations. The authors have cited additional references within the Supporting Information.^[^^32–43^^]^

## Conflict of Interest

The authors declare no conflict of interest.

## Supporting information

Supplementary Material

## Data Availability

The data that support the findings of this study are available from the corresponding author upon reasonable request.

## References

[1] T. Kubota , H. J. Miyazaki , Bull. Chem. Soc. Jpn. 1962, 35, 1549.

[2] G. Anthoine , J. Nasielski , E. Van der Donckt , N. Vanlautem , Bull. Soc. Chim. Belg. 1967, 76, 230.

[3] D. Cantin , J.‐M. Richard , J. Alary , Electrochim. Acta 1988, 33, 1047.

[4] M. Ruiz Montoya , J. M. Rodriguez Mellado , R. M. Galvin , J. Electroanal. Chem. 1990, 293, 185.

[5] Y. Wang , L. Zhang , Synthesis 2015, 47, 289.

[6] K. D. Kim , J. H. Lee , Org. Lett. 2018, 20, 7712. 30481038 10.1021/acs.orglett.8b03446

[7] J. Singh , R. I. Patel , A. Sharma , Adv. Synth. Catal. 2022, 364, 2289.

[8] M. Kjellberg , A. Ohleier , P. Thuéry , E. Nicolas , L. Anthore‐Dalion , T. Cantat , Chem. Sci. 2021, 12, 10266. 34377414 10.1039/d1sc01974k PMC8336470

[9] M. O. Konev , L. Cardinale , A. von Wangelin , Org. Lett. 2020, 22, 1316. 31967477 10.1021/acs.orglett.9b04632

[10] S. H. Kim , J. H. An , J. H. Lee , Org. Biomol. Chem. 2021, 19, 3755.

[11] P. Xu , H.‐C. Xu , Synlett 2019, 30, 1219.

[12] Y. Fukazawa , Y. Rubtsov , A. V. Markov , Eur. J. Org. Chem. 2020, 2020, 3317.

[13] H. Miyazaki , Y. Matshuhisa , T. Kubota , Bull. Chem. Soc. Jpn. 1981, 54, 3850.

[14] M. L. Pegis , B. A. McKeown , N. Kumar , K. Lang , D. J. Wasylenko , X. P. Zhang , S. Raugei , ACS Cent. Sci. 2016, 2, 850. 27924314 10.1021/acscentsci.6b00261 PMC5126711

[15] C. Amatore , G. Capobianco , G. Farina , G. Sadona , J.‐M. Savéant , M.‐G. Severin , E. Vianello , J. Am. Chem. Soc. 1985, 107, 1815.

[16] L. Chmurzynski , L. Pawlak , H. Myska , J. Mol. Struct. 1982, 80, 235.

[17] A. S. Kurbatova , Y. V. Kurbatov , Khim. Geterotsikl. Soedin. 1988, 1, 133.

[18] G. A. N. Felton , A. K. Vannucci , N. Okumura , L. T. Lockett , D. H. Evans , R. S. Glass , D. L. Lichtenberger , Organometallics 2008, 27, 4671.

[19] L. Chmurzynski , Anal. Chim. Acta 1996, 321, 237.

[20] C. Costentin , C. Louault , M. Robert , J.‐M. Savéant , Proc. Natl. Acad. Sci. 2009, 106, 18143. 19822746 10.1073/pnas.0910065106 PMC2775288

[21] C. Costentin , M. Robert , J.‐M. Savéant , J. Am. Chem. Soc. 2003, 125, 105. 12515511 10.1021/ja027287f

[22] J.‐M. Savéant , C. Costentin , Elements of Molecular and Biomolecular Electrochemistry, 2nd ed., VCH, Wiley, Hoboken NJ 2019.

[23] C. E. D. Chidsey , Science 1991, 251, 919. 17847385 10.1126/science.251.4996.919

[24] P. Ballinger , F. A. Long , J. Am. Chem. Soc. 1960, 82, 795.

[25] E. Rossini , A. D. Bochevarov , E. W. Knapp , ACS Omega 2018, 3, 1653. 31458485 10.1021/acsomega.7b01895 PMC6641400

[26] R. W. Taft , M. J. Kamlet , J. Am. Chem. Soc. 1976, 98, 2286.

[27] N. Nishi , K. Yamamoto , H. Shinohara , U. Nagashima , T. Okuyama , Chem. Phys. Lett. 1985, 122, 599.

[28] A. Elangovan , R. Shanmugan , G. Arivazhagan , A. Mahendraprabu , N. K. Karthick , Chem. Phys. Lett. 2015, 639, 161.

[29] J. M. Savéant , J. Am. Chem. Soc. 1987, 109, 6788.

[30] C. P. Andrieux , E. Differding , M. Robert , J. M. Savéant , J. Am. Chem. Soc. 1993, 115, 6592.

[31] M. A. Elsayed , Desalin. Water Treat. 2023, 53, 57.

