# Supplementary material for: Proton‐Coupled Electron Transfer Deoxygenation of Pyridine N‐Oxide: A Mechanistic Study
Source: Chemphyschem. 2025 Sep 21;26(21):e202500292. doi: 10.1002/cphc.202500292 (PMC12597217; doi:10.1002/cphc.202500292)
Supplement: Supplementary file 1 — Supplementary Material [file CPHC-26-e202500292-s001.zip › cphc202500292-sup-0001-SuppData-S1/cphc202500292-sup-0001-SuppData-S1.pdf]

Supporting Information

Proton-Coupled Electron Transfer Deoxygenation of Pyridine N-oxide: A Mechanistic Study

Céline Naddour, Gabriel Durin, Sylvie Chardon-Noblat, Cyrille Costentin*

[a] Univ Grenoble Alpes, DCM, CNRS, 38000 Grenoble, France.

cyrille.costentin@univ-grenoble-alpes.fr

Table of Content

1. Additional CV of PNO S3

2. Determination of C_d and R_u. S4

3. Determination of the diffusion coefficient and the standard potential of PNO S5

4. Ruling out a simple parent-child chemical step following electron transfer (and other mechanisms) S6

5. Derivation of equations S8

5.1. Mechanism without added proton source

5.2. Mechanism with an added proton source (AH)

6. Simulations of CVs S18

6.1. Simulation parameters

6.2. PNO 1.15 mM no added water

6.3. PNO 1.15 mM addition of water

6.4. PNO 1 mM no added water

6.5. PNO 1 mM addition of EtOH

7. Constant potential electrolysis without added acid S24

8. CVs of PNO and Py in the presence of water S25

9. Constant potential electrolysis with added water S26

10. DFT calculations S27

10. 1 General consideration

10.2 Three lowest frequencies and Gibbs free energy for all computed structures

11. References S29

1. Additional CV of PNO

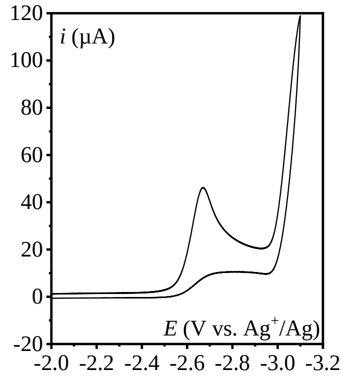

Fig. S1. CV of PNO at 0.1 V/s on a 3 mm diameter GCE in CH_3CN + 0.1 M Bu_4NPF_6 under Ar.

2. Determination of C_d and R_u.

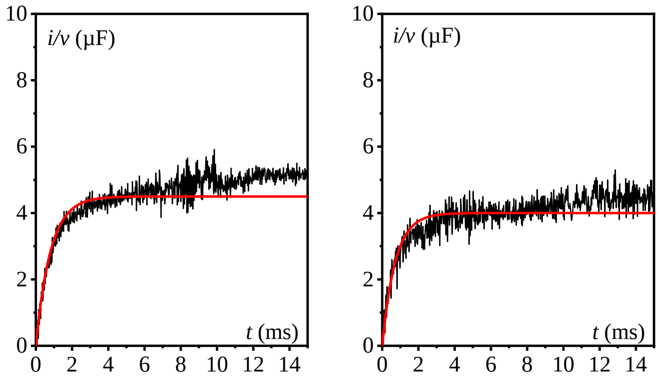

Fig. S2. Charging of the capacitance of a 3 mm diameter GCE in CH_3CN + 0.1 M Bu_4NPF_6 under Ar starting at -1 V vs. Ag^+/Ag and scanning in the cathodic direction at 45 V/s. Red: fitting with $\frac{i}{v} = C_d\left[1 - \exp\left(-\frac{t}{R_u C_d}\right)\right]$. (Left and right figures correspond to two different experiments).

We obtain: $C_d = 4$ μF and $R_u = 180$ Ω.

3. Determination of the diffusion coefficient and the standard potential of PNO

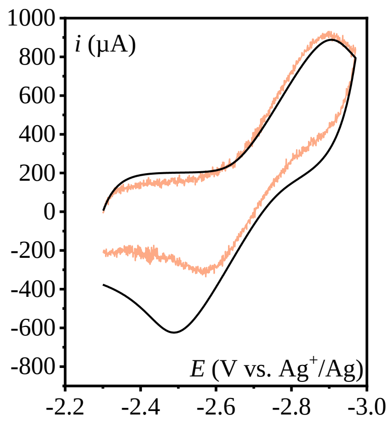

Fig. S3. Orange: CV of 1 mM PNO at 45 V/s in CH_3CN + 0.1 M *n*-Bu_4NPF_6 under Ar on a 3 mm diameter GCE. Black line: simulation of a reversible nernstian monoelectronic couple at 45 V/s, 1 mM, with $D = 6\ 10^{-5}$ cm^2/s, $E^0 = 2.675$ V vs. Ag^+/Ag, R_u = 180 Ω and C_d = 4.5 µF, T = 298 K and S = 0.07 cm^2.

4. Ruling out a simple parent-child chemical step following electron transfer (and other mechanisms)

We consider a simple parent-child mechanism as depicted in Scheme S1. The system is controlled by a dimensionless parameter: $\lambda_{RS} = \dfrac{k_{RS}C^0}{Fv/RT}$. In Figures S4 are reported the theoretical working curve obtained from simulations using Digielch of the ratio $i_{p,ox}/i_{p,red}$ and the ratio $i_{p,red}/i_p^0$ for such a mechanism, where $i_{p,ox}$ and $i_{p,red}$ are the anodic and cathodic peak currents and i_p^0 the corresponding Randles-Sevcik current, as function of $\log\lambda_{RS}$. Simulations have been performed with the potential inversion being 0.3 V more negative than E^0. Adjustment of experimental data recorded with PNO 1 mM in CH_3CN + 0.1 M *n*-Bu_4NPF_6 under Ar on a 3 mm diameter GCE at scan rates from 10 to 45 V/s would lead to $k = 7\ 10^5$ $M^{-1}s^{-1}$ (Figure S4a). However, considering this rate constant it can be seen in Figure S4b that the decrease of the stoichiometry of the reaction would occur at a much larger rate than observed (data points in Figure S4b correspond to scan rate from 10 to 0.05 V/s). Similarly, neither a simple "radical-radical" dimerization ($\lambda_{RR} = \dfrac{k_{RR}C^0}{Fv/RT}$, Figures S4c-d) or a simple EC mechanism ($\lambda_{EC} = \dfrac{k_{EC}}{Fv/RT}$, Figures S4e-f) or a radical-substrate followed by a reduction (RS-DISP-ECE) ($\lambda_{RS-dispece} = \dfrac{k_{RS}C^0}{Fv/RT}$, Figures S4g-h) can account for the experimental data as the stoichiometry would not get below one.

Scheme S1

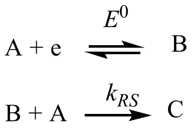

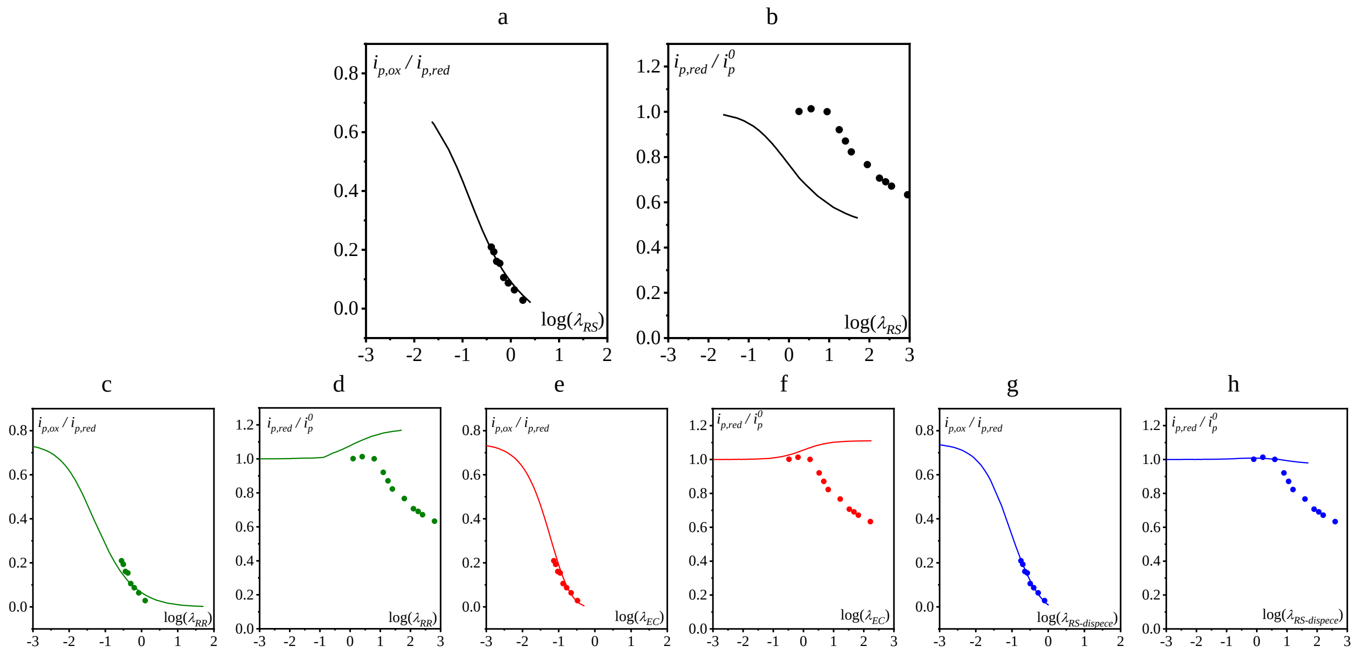

Fig. S4. Working curves (full lines obtained from simulations using Digielch program) and experimental data (points) for the evolution of $i_{p,ox}/i_{p,red}$ and $i_{p,red}/i_p^0$ as function of the dimensionless parameter for various mechanisms following an initial electron transfer. (a) and (b) Parent-child reaction (scheme S1). (c) and (d) Radical-radical dimerization. (e) and (f) radical-substrate

followed by a reduction (RS-DISP-ECE). (g) and (h) first order reaction. In all case, the rate constant governing the mechanism is adjusted for $i_{p,ox} / i_{p,red}$ to match with the working curve and the same rate constant is used to plot the $i_{p,red} / i_p^0$ evolution.

5. Derivation of equations

5.1. Mechanism without added proton source

We consider the mechanism depicted in scheme S2.

Scheme S2

$$\mathrm{A} + \mathrm{e} \underset{}{\overset{E^0}{\rightleftharpoons}} \mathrm{B}$$

$$\mathrm{B} \xrightarrow{k_1} \mathrm{C}$$

$$\mathrm{C} + \mathrm{A} \xrightarrow{k_2} \mathrm{D}$$

Formulation:

The electrode potential E is swept linearly between two values E_i and E_f :

$0 \le t \le t_R : E = E_i - vt$, $t_R \le t \le 2t_R : E = E_f - v(t - t_R)$ (t_R is the time at which the linear potential is reversed)

$\forall t,\ x = \infty$ and $\forall x,\ t = 0$:

$[\mathrm{A}]_{bulk} = C_{\mathrm{A}}^0$; $[\mathrm{B}]_{bulk} = [\mathrm{C}]_{bulk} = [\mathrm{D}]_{bulk} = 0$

$t > 0,\ 0 < x < \infty$:

For the sake of simplicity, we assume that all species have the same diffusion coefficient.

$$\frac{\partial[\mathrm{A}]}{\partial t} = D\frac{\partial^2[\mathrm{A}]}{\partial x^2} - k_2[\mathrm{A}][\mathrm{C}]$$

$$\frac{\partial[\mathrm{B}]}{\partial t} = D\frac{\partial^2[\mathrm{B}]}{\partial x^2} - k_1[\mathrm{B}]$$

$$\frac{\partial[\mathrm{C}]}{\partial t} = D\frac{\partial^2[\mathrm{C}]}{\partial x^2} + k_1[\mathrm{B}] - k_2[\mathrm{A}][\mathrm{C}]$$

$$\frac{\partial[\mathrm{D}]}{\partial t} = D\frac{\partial^2[\mathrm{D}]}{\partial x^2} + k_2[\mathrm{A}][\mathrm{C}]$$

$x = 0$:

$$\frac{i}{FS} = D\left(\frac{\partial[\mathrm{A}]}{\partial x}\right)_{x=0} = -D\left(\frac{\partial[\mathrm{B}]}{\partial x}\right)_{x=0}$$

$$\left(\frac{\partial[\mathrm{C}]}{\partial x}\right)_{x=0} = \left(\frac{\partial[\mathrm{D}]}{\partial x}\right)_{x=0} = 0$$

Fast electron transfer:

$$\frac{[\mathrm{B}]_0}{[\mathrm{A}]_0} = \exp\left[-\frac{F}{RT}\left(E - E^0\right)\right]$$

We introduce dimensionless parameters:

$$\xi = -\frac{F}{RT}\left(E - E^0\right);\ \psi = \frac{i}{FSC_{\mathrm{A}}^0\sqrt{D}\sqrt{\frac{Fv}{RT}}};\ a = \frac{[\mathrm{A}]}{C_{\mathrm{A}}^0};\ b = \frac{[\mathrm{B}]}{C_{\mathrm{A}}^0};\ c = \frac{[\mathrm{C}]}{C_{\mathrm{A}}^0};\ d = \frac{[\mathrm{D}]}{C_{\mathrm{A}}^0};\ \tau = \frac{Fv}{RT}t;\ y = x\sqrt{\frac{Fv}{DRT}};\ \lambda_1 = \frac{k_1}{Fv/RT};$$

$$\lambda_2 = \frac{k_2 C_{\mathrm{A}}^0}{Fv/RT}$$

Formulation

$\forall\,\tau,\ y = \infty$ and $\forall y,\ \tau = 0$:

$a = 1$ and $b = c = d = 0$

$\tau > 0,\ 0 < y < \infty$:

$$\frac{\partial a}{\partial \tau} = \frac{\partial^2 a}{\partial y^2} - \lambda_2 a \times c$$

$$\frac{\partial b}{\partial \tau} = \frac{\partial^2 b}{\partial y^2} - \lambda_1 b$$

$$\frac{\partial c}{\partial \tau} = \frac{\partial^2 c}{\partial y^2} + \lambda_1 b - \lambda_2 a \times c$$

$$\frac{\partial d}{\partial \tau} = \frac{\partial^2 d}{\partial y^2} + \lambda_2 a \times c$$

$y = 0$:

$$\psi = \left(\frac{\partial a}{\partial y}\right)_0 = -\left(\frac{\partial b}{\partial y}\right)_0$$

$$\left(\frac{\partial c}{\partial y}\right)_0 = \left(\frac{\partial d}{\partial y}\right)_0 = 0$$

$$\frac{b_0}{a_0} = \exp(\xi)$$

The system is fully controlled by two parameters $\lambda_1 = \frac{k_1}{Fv/RT}$ and $\lambda_2 = \frac{k_2 C_{\mathrm{A}}^0}{Fv/RT}$.

Resolution

We have $\frac{\partial(a+b+c+2d)}{\partial \tau} = \frac{\partial^2(a+b+c+2d)}{\partial y^2}$ which resolution together with boundary conditions, gives:

$$a_0 + b_0 + c_0 + 2d_0 = 1$$

Resolution of $\frac{\partial b}{\partial \tau} = \frac{\partial^2 b}{\partial y^2} - \lambda_1 b$, together with boundary conditions, gives: $b_0 = \frac{1}{\sqrt{\pi}}\int_0^{\tau}\frac{\exp\left[-\lambda_1(\tau-\eta)\right]\times\psi}{\sqrt{\tau-\eta}}d\eta$

We also have: $\frac{\partial(b+c+d)}{\partial \tau} = \frac{\partial^2(b+c+d)}{\partial y^2}$ which resolution together with boundary conditions, gives:

$$b_0 + c_0 + d_0 = \frac{1}{\sqrt{\pi}} \int_0^{\tau} \frac{\psi}{\sqrt{\tau - \eta}} d\eta$$

To get a full resolution an additional equation is required. Due to the non-linear character of the diffusion-reaction differential equation on A, C and D there is no analytical solution in the general case. Nonetheless we can derive the competition parameter controlling the kinetic competition between steps 1 and 2.

We introduce: $a^* = a\sqrt{\lambda_1}$; $b^* = b\sqrt{\lambda_1}$; $b^* = b\sqrt{\lambda_1}$; $y^* = y\sqrt{\lambda_1}$; $\tau^* = \lambda_1 \tau$ leading to:

$$\frac{\partial a^*}{\partial \tau^*} = \frac{\partial^2 a^*}{\partial y^{*2}} - \frac{\lambda_2}{\lambda_1^{3/2}} a^* \times c^*$$

$$\frac{\partial b^*}{\partial \tau^*} = \frac{\partial^2 b^*}{\partial y^{*2}} - b^*$$

$$\frac{\partial c^*}{\partial \tau^*} = \frac{\partial^2 c^*}{\partial y^{*2}} + b^* - \frac{\lambda_2}{\lambda_1^{3/2}} a^* \times c^*$$

This shows that the competition parameter is $p = \frac{\lambda_2}{\lambda_1^{3/2}} = \frac{k_2 C_{\mathrm{A}}^0}{k_1^{3/2}} \left(\frac{Fv}{RT} \right)^{1/2}$.

A zone diagram can be obtained sketching the kinetic behavior of the system as function of the two dimensionless parameters $\lambda_1 = \frac{k_1}{Fv/RT}$ and $\lambda_2 = \frac{k_2 C_{\mathrm{A}}^0}{Fv/RT}$. We first derive limiting cases.

Limiting cases:

1. $\lambda_2 \to 0$: EC mechanism

When $\lambda_1 \to 0$, a reversible one electron nernstian wave is obtained (DO zone)

When $\lambda_1 \to \infty$, an irreversible one electron nernstian wave is obtained (KP_1 zone) with:

$$E_p = E^0 - 0.78 \frac{RT}{F} + \frac{RT}{2F} \ln\left(k_1 \frac{RT}{Fv} \right) \text{ and } i_p = 0.496 FSC_{\mathrm{A}}^0 \sqrt{DFv/RT}$$

2. $\lambda_2 \to \infty$: Steady-state approximation for C: $\lambda_1 b = \lambda_2 a \times c$

$$\frac{\partial (a-b)}{\partial \tau} = \frac{\partial^2 (a-b)}{\partial y^2} \text{ hence } a_0 - b_0 = 1 - 2I_{\psi}$$

Thus: $\left[\frac{1}{\sqrt{\pi}} \int_0^{\tau} \frac{\exp\left[-\lambda_1 (\tau - \eta) \right] \times \psi}{\sqrt{\tau - \eta}} d\eta \right] \times \left[\exp(-\xi) - 1 \right] = 1 - 2I_{\psi}$ corresponding to the $KO_{0.5}$ zone)

When $\lambda_1 \to \infty$, $\left[\frac{1}{\sqrt{\pi}} \int_0^{\tau} \frac{\exp\left[-\lambda_1(\tau-\eta)\right]\times\psi}{\sqrt{\tau-\eta}} d\eta \right] \to \frac{\psi}{\sqrt{\lambda_1}}$, an irreversible one electron nernstian wave is obtained (KP$_1$ zone) with:

$$E_p = E^0 - 0.78\frac{RT}{F} + \frac{RT}{2F}\ln\left(4k_1\frac{RT}{Fv}\right) \text{ and } i_p = \frac{0.496}{2} FSC_A^0 \sqrt{DFv/RT}$$

When $\lambda_1 \to 0$, $\left[\frac{1}{\sqrt{\pi}} \int_0^{\tau} \frac{\exp\left[-\lambda_1(\tau-\eta)\right]\times\psi}{\sqrt{\tau-\eta}} d\eta \right] \to I_\psi$, a reversible one electron nernstian wave is obtained (DO zone).

3. $\lambda_1 \to \infty$: pure kinetics conditions for B:

We have 4 equations, $b_0 = \frac{\psi}{\sqrt{\lambda_1}}$; $a_0 + b_0 + c_0 + 2d_0 = 1$; $b_0 = a_0 \exp(\xi)$; $a_0 + d_0 = 1 - \frac{1}{\sqrt{\pi}} \int_0^{\tau} \frac{\psi}{\sqrt{\tau-\eta}} d\eta$

A fifth equation is required; as already mentioned, due to the non-linearity of the system, there is no analytical solution but the governing parameter is *p*.

When $p \to \infty$: Steady-state approximation for C: $b^* = p \times a^* \times c^*$

$\frac{\partial(a^*-b^*)}{\partial \tau^*} = \frac{\partial^2(a^*-b^*)}{\partial y^{*2}}$ hence $a_0 - b_0 = 1 - 2I_\psi$

Thus: $\frac{\psi}{\sqrt{\lambda_1}}\left[\exp(-\xi)-1\right] = 1 - 2I_\psi$

Because B is at steady state, its concentration remains small and therefore: $\frac{\psi}{\sqrt{\lambda_1}}\exp(-\xi) = 1 - 2I_\psi$. Introducing $\psi' = 2\psi$ we have:

$\psi' \exp\left(-\xi - \ln\sqrt{4\lambda_1}\right) = 1 - I_{\psi'}$ and therefore the wave corresponds to an irreversible wave (KP$_{0.5}$ zone) with:

$$E_p = E^0 - 0.78\frac{RT}{F} + \frac{RT}{2F}\ln\left(4k_1\frac{RT}{Fv}\right) \text{ and } i_p = \frac{0.496}{2} FSC_A^0 \sqrt{DFv/RT}$$

When $p \to 0$: $a_0 = 1 - I_\psi$, back to the KP$_1$ zone.

4. $\lambda_1 \to 0$: $b_0 = \frac{1}{\sqrt{\pi}} \int_0^{\tau} \frac{\exp\left[-\lambda_1(\tau-\eta)\right]\times\psi}{\sqrt{\tau-\eta}} d\eta \to I_\psi$; thus $c_0 + d_0 = 0$ and in fact $c_0 = d_0 = 0$. Hence, $\forall p$, a reversible one electron nernstian wave is obtained (DO zone).

The zone diagram is shown in figure S5.

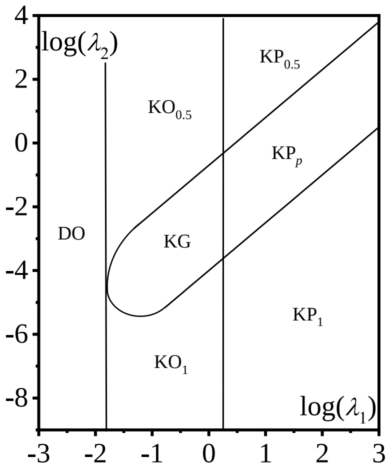

Fig. S5. Zone diagram corresponding to the mechanism depicted in scheme S2.

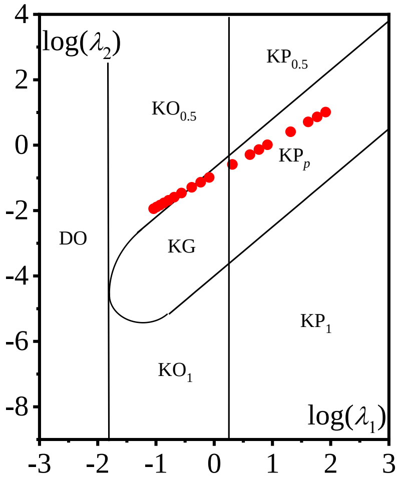

Fig. S6. Zone diagram corresponding to the mechanism depicted in scheme S2 with, in red, positions of the system ([PNO] = 1 mM) as function of the scan rate (v = 0.05 to 45 V/s) with the rate constant evaluated from the CV analysis: $k_1 = 160\ s^{-1}$ and $k_2 = 2\ 10^4\ M^{-1}s^{-1}$.

5.2. Mechanism with an added proton source (AH)

5.2.1. We consider the mechanism depicted in scheme S3 corresponding to a stepwise reductive cleavage of C

Scheme S3

$$\mathrm{A + e} \underset{}{\overset{E^0}{\rightleftharpoons}} \mathrm{B}$$

$$\mathrm{B} \xrightarrow{k_1(\mathrm{AH})} \mathrm{C}$$

$$\mathrm{C + A} \xrightarrow{k_2} \mathrm{D}$$

$$\mathrm{C} \xrightarrow{k_{\mathrm{hom}}(\mathrm{AH})} \mathrm{F} + \text{other group}$$

$$\mathrm{F + e} \underset{}{\overset{E^0{}_2 >> E^0}{\rightleftharpoons}} \mathrm{G}$$

We do not consider the disproportionation reaction between F and B because F is produced in a space where $[\mathrm{B}] \approx 0$. Additionally, we make the assumption that k_2 is independent of the proton donor.

Formulation:

The electrode potential E is swept linearly between two values E_i and E_f :

$0 \le t \le t_R : E = E_i - vt$, $t_R \le t \le 2t_R : E = E_f - v(t - t_R)$ (t_R is the time at which the linear potential is reversed)

$\forall t,\ x = \infty$ and $\forall x,\ t = 0$:

$[\mathrm{A}]_{bulk} = C^0_{\mathrm{A}}$; $[\mathrm{B}]_{bulk} = [\mathrm{C}]_{bulk} = [\mathrm{D}]_{bulk} = [\mathrm{F}]_{bulk} = [\mathrm{G}]_{bulk} = 0$

$t > 0,\ 0 < x < \infty$:

For the sake of simplicity, we assume that all species have the same diffusion coefficient.

$$\frac{\partial[\mathrm{A}]}{\partial t} = D\frac{\partial^2[\mathrm{A}]}{\partial x^2} - k_2[\mathrm{A}][\mathrm{C}]$$

$$\frac{\partial[\mathrm{B}]}{\partial t} = D\frac{\partial^2[\mathrm{B}]}{\partial x^2} - k_1[\mathrm{B}]$$

$$\frac{\partial[\mathrm{C}]}{\partial t} = D\frac{\partial^2[\mathrm{C}]}{\partial x^2} + k_1[\mathrm{B}] - k_2[\mathrm{A}][\mathrm{C}] - k_{\mathrm{hom}}[\mathrm{C}]$$

$$\frac{\partial[\mathrm{D}]}{\partial t} = D\frac{\partial^2[\mathrm{D}]}{\partial x^2} + k_2[\mathrm{A}][\mathrm{C}]$$

$$\frac{\partial[\mathrm{F}]}{\partial t} = D\frac{\partial^2[\mathrm{F}]}{\partial x^2} + k_C[\mathrm{C}]$$

$$\frac{\partial[\mathrm{G}]}{\partial t} = D\frac{\partial^2[\mathrm{G}]}{\partial x^2}$$

$x = 0$:

$$\frac{i_1}{FS} = D\left(\frac{\partial[\mathrm{A}]}{\partial x}\right)_{x=0} = -D\left(\frac{\partial[\mathrm{B}]}{\partial x}\right)_{x=0}$$

$$\frac{i_2}{FS} = D\left(\frac{\partial[\mathrm{F}]}{\partial x}\right)_{x=0} = -D\left(\frac{\partial[\mathrm{G}]}{\partial x}\right)_{x=0}$$

$$i = i_1 + i_2$$

$$\left(\frac{\partial[\mathrm{C}]}{\partial x}\right)_{x=0}=\left(\frac{\partial[\mathrm{D}]}{\partial x}\right)_{x=0}=0$$

Fast electron transfer:

$$\frac{[\mathrm{B}]_0}{[\mathrm{A}]_0}=\exp\left[-\frac{F}{RT}\left(E-E^0\right)\right]$$

$$\frac{[\mathrm{G}]_0}{[\mathrm{F}]_0}=\exp\left[-\frac{F}{RT}\left(E-E_2^0\right)\right]$$

We introduce dimensionless parameters:

$$\xi=-\frac{F}{RT}\left(E-E^0\right);\ \psi=\frac{i}{FSC_\mathrm{A}^0\sqrt{D}\sqrt{\frac{Fv}{RT}}};\ a=\frac{[\mathrm{A}]}{C_\mathrm{A}^0}\sqrt{\lambda_1};\ b=\frac{[\mathrm{B}]}{C_\mathrm{A}^0}\sqrt{\lambda_1};\ c=\frac{[\mathrm{C}]}{C_\mathrm{A}^0}\sqrt{\lambda_1};\ d=\frac{[\mathrm{D}]}{C_\mathrm{A}^0}\sqrt{\lambda_1};\ f=\frac{[\mathrm{F}]}{C_\mathrm{A}^0}\sqrt{\lambda_1};\ g=\frac{[\mathrm{G}]}{C_\mathrm{A}^0}\sqrt{\lambda_1}$$

$$;\ \tau^*=k_1t;\ y^*=x\sqrt{\frac{k_1}{D}};\ \lambda_1=\frac{k_1}{Fv/RT};\ \lambda_2=\frac{k_2C_\mathrm{A}^0}{Fv/RT};\ \lambda_\mathrm{hom}=\frac{k_\mathrm{hom}}{Fv/RT};\ \Delta\xi^0=\frac{F}{RT}\left(E_2^0-E^0\right)$$

Formulation

$\forall\,\tau^*,\ y^*=\infty$ and $\forall y^*,\ \tau^*=0$:

$a^*=\sqrt{\lambda_1}$ and $b^*=c^*=d^*=f^*=g^*=0$

$\tau^*>0,\ 0<y^*<\infty$:

$$\frac{\partial a^*}{\partial\tau^*}=\frac{\partial^2a^*}{\partial y^{*2}}-\frac{\lambda_2}{\lambda_1^{3/2}}a^*\times c^*$$

$$\frac{\partial b^*}{\partial\tau^*}=\frac{\partial^2b^*}{\partial y^{*2}}-b^*$$

$$\frac{\partial c^*}{\partial\tau^*}=\frac{\partial^2c^*}{\partial y^{*2}}+b^*-\frac{\lambda_2}{\lambda_1^{3/2}}a^*\times c^*$$

$$\frac{\partial d^*}{\partial\tau^*}=\frac{\partial^2d^*}{\partial y^{*2}}+\frac{\lambda_2}{\lambda_1^{3/2}}a^*\times c^*$$

$$\frac{\partial f^*}{\partial\tau^*}=\frac{\partial^2f^*}{\partial y^{*2}}+\frac{\lambda_\mathrm{hom}}{\lambda_1}c^*$$

$$\frac{\partial g^*}{\partial\tau^*}=\frac{\partial^2g^*}{\partial y^{*2}}$$

$y^*=0$:

$$\psi_1=\left(\frac{\partial a^*}{\partial y^*}\right)_0=-\left(\frac{\partial b^*}{\partial y^*}\right)_0$$

$$\psi_2=\left(\frac{\partial f^*}{\partial y^*}\right)_0=-\left(\frac{\partial g^*}{\partial y^*}\right)_0$$

$$\psi=\psi_1+\psi_2$$

$$\left(\frac{\partial c^*}{\partial y^*}\right)_0 = \left(\frac{\partial d^*}{\partial y^*}\right)_0 = 0$$

$$\frac{b_0^*}{a_0^*} = \exp(\xi)$$

$$\frac{g_0^*}{f_0^*} = \exp\left(\xi + \Delta\xi^0\right)$$

The system is fully controlled by two parameters $\frac{\lambda_{\text{hom}}}{\lambda_1}$ and $\frac{\lambda_2}{\lambda_1^{3/2}}$ (taking into account that $\Delta\xi^0 >> 0$).

5.2.2. We consider the mechanism depicted in scheme S4 corresponding to a concerted dissociative reduction of C

Scheme S4

$$\mathrm{A + e} \underset{}{\overset{E^0}{\rightleftharpoons}} \mathrm{B}$$

$$\mathrm{B} \xrightarrow{k_1(\mathrm{AH})} \mathrm{C}$$

$$\mathrm{C + A} \xrightarrow{k_2} \mathrm{D}$$

$$\mathrm{C + e} \xrightarrow[k_\mathrm{S}(\mathrm{AH})]{E^0_{\mathrm{DET}}>>E^0} \mathrm{G + leaving\ group}$$

We make the assumption that k_2 is independent of the proton donor.

Formulation:

The electrode potential E is swept linearly between two values E_i and E_f :

$0 \le t \le t_R : E = E_i - vt$, $t_R \le t \le 2t_R : E = E_f - v(t - t_R)$ (t_R is the time at which the linear potential is reversed)

$\forall t,\ x = \infty$ and $\forall x,\ t = 0$:

$[\mathrm{A}]_{bulk} = C_\mathrm{A}^0$; $[\mathrm{B}]_{bulk} = [\mathrm{C}]_{bulk} = [\mathrm{D}]_{bulk} = [\mathrm{G}]_{bulk} = 0$

$t > 0,\ 0 < x < \infty$:

For the sake of simplicity, we assume that all species have the same diffusion coefficient.

$$\frac{\partial[\mathrm{A}]}{\partial t} = D\frac{\partial^2[\mathrm{A}]}{\partial x^2} - k_2[\mathrm{A}][\mathrm{C}]$$

$$\frac{\partial[\mathrm{B}]}{\partial t} = D\frac{\partial^2[\mathrm{B}]}{\partial x^2} - k_1[\mathrm{B}]$$

$$\frac{\partial[\mathrm{C}]}{\partial t} = D\frac{\partial^2[\mathrm{C}]}{\partial x^2} + k_1[\mathrm{B}] - k_2[\mathrm{A}][\mathrm{C}]$$

$$\frac{\partial[\mathrm{D}]}{\partial t} = D\frac{\partial^2[\mathrm{D}]}{\partial x^2} + k_2[\mathrm{A}][\mathrm{C}]$$

$$\frac{\partial[\mathrm{G}]}{\partial t} = D\frac{\partial^2[\mathrm{G}]}{\partial x^2}$$

$x = 0$:

$$\frac{i_1}{FS} = D\left(\frac{\partial[\mathrm{A}]}{\partial x}\right)_{x=0} = -D\left(\frac{\partial[\mathrm{B}]}{\partial x}\right)_{x=0}$$

$$\frac{i_2}{FS} = D\left(\frac{\partial[\mathrm{C}]}{\partial x}\right)_{x=0}$$

$$i = i_1 + i_2$$

$$\left(\frac{\partial[\mathrm{D}]}{\partial x}\right)_{x=0} = 0$$

Fast electron transfer:

$$\frac{[\mathrm{B}]_0}{[\mathrm{A}]_0} = \exp\left[-\frac{F}{RT}\left(E - E^0\right)\right]$$

DET kinetics

$$i_2 = FS[\mathrm{C}]_0\, k_S \exp\left[-\frac{\alpha F}{RT}\left(E - E^0_{DET}\right)\right]$$

We introduce dimensionless parameters:

$$\xi = -\frac{F}{RT}\left(E - E^0\right);\ \psi = \frac{i}{FSC^0_{\mathrm{A}}\sqrt{D}\sqrt{\frac{Fv}{RT}}};\ a = \frac{[\mathrm{A}]}{C^0_{\mathrm{A}}}\sqrt{\lambda_1};\ b = \frac{[\mathrm{B}]}{C^0_{\mathrm{A}}}\sqrt{\lambda_1};\ c = \frac{[\mathrm{C}]}{C^0_{\mathrm{A}}}\sqrt{\lambda_1};\ d = \frac{[\mathrm{D}]}{C^0_{\mathrm{A}}}\sqrt{\lambda_1};\ g = \frac{[\mathrm{G}]}{C^0_{\mathrm{A}}}\sqrt{\lambda_1};\ \tau^* = k_1 t;$$

$$y^* = x\sqrt{\frac{k_1}{D}};\ \lambda_1 = \frac{k_1}{Fv/RT};\ \lambda_2 = \frac{k_2 C^0_{\mathrm{A}}}{Fv/RT};\ \Delta\xi^0 = \frac{F}{RT}\left(E^0_{DET} - E^0\right);\ \Lambda = \frac{k_S}{\sqrt{Dk_1}}$$

Formulation

$\forall\, \tau^*,\ y^* = \infty$ and $\forall y^*,\ \tau^* = 0$:

$a^* = \sqrt{\lambda_1}$ and $b^* = c^* = d^* = f^* = g^* = 0$

$\tau^* > 0,\ 0 < y^* < \infty$:

$$\frac{\partial a^*}{\partial \tau^*} = \frac{\partial^2 a^*}{\partial y^{*2}} - \frac{\lambda_2}{\lambda_1^{3/2}} a^* \times c^*$$

$$\frac{\partial b^*}{\partial \tau^*} = \frac{\partial^2 b^*}{\partial y^{*2}} - b^*$$

$$\frac{\partial c^*}{\partial \tau^*} = \frac{\partial^2 c^*}{\partial y^{*2}} + b^* - \frac{\lambda_2}{\lambda_1^{3/2}} a^* \times c^*$$

$$\frac{\partial d^*}{\partial \tau^*} = \frac{\partial^2 d^*}{\partial y^{*2}} + \frac{\lambda_2}{\lambda_1^{3/2}} a^* \times c^*$$

$$\frac{\partial g^*}{\partial \tau^*} = \frac{\partial^2 g^*}{\partial y^{*2}}$$

$y^* = 0$:

$$\psi_1 = \left(\frac{\partial a^*}{\partial y^*}\right)_0 = -\left(\frac{\partial b^*}{\partial y^*}\right)_0$$

$$\psi_2 = \left(\frac{\partial c^*}{\partial y^*}\right)_0$$

$$\psi = \psi_1 + \psi_2$$

$$\left(\frac{\partial d^*}{\partial y^*}\right)_0 = 0$$

$$\frac{b_0^*}{a_0^*} = \exp(\xi)$$

$$\psi_2 = \Lambda c_0^* \exp\left[\alpha\left(\xi + \Delta\xi^0\right)\right]$$

The system is fully controlled by two parameters $\Lambda = \frac{k_S}{\sqrt{Dk_1}}$ and $\frac{\lambda_2}{\lambda_1^{3/2}}$ (taking into account that $\Delta\xi^0$ and α are known).

6. Simulations of CVs

All simulations are performed with DigiElch program.

6.1. Simulation parameters

All CVs were simulated considering a semi-infinite linear diffusion with D = 6 10^{-5} cm^2/s (for all species), S = 0.07 cm^2, T = 298 K.

C_d was adjusted between 4 to 6 µF depending on the experimental data. The uncompensated resistance R_u was 80 Ω for scan rates between 0.05 and 7 V/s and 180 Ω for scan rates between 10 to 45 V/s.

The initial, inversion and final potentials are adjusted to experimental conditions.

"HOM" mechanism

$$\mathrm{A + e} \underset{}{\overset{E^0}{\rightleftharpoons}} \mathrm{B}$$

$$\mathrm{B} \xrightarrow{k_1(\mathrm{AH})} \mathrm{C}$$

$$\mathrm{C + A} \xrightarrow{k_2} \mathrm{D}$$

$$\mathrm{C} \xrightarrow{k_{\mathrm{hom}}(\mathrm{AH})} \mathrm{F + other\ group}$$

$$\mathrm{F + e} \overset{E^0_3 >> E^0}{\rightleftharpoons} \mathrm{G}$$

$$\mathrm{F + B} \xrightarrow{k_{\mathrm{dif}}} \mathrm{G + A}$$

A/B electron transfer: E^0 = -2.675 V, $k_S = 10^4$ cm/s (Nernstian)

B/C reaction: K_1 = large (no effect as soon as K_1 >10), $k_1 =$ 160 s^{-1} without proton donor and adjusted in the presence of proton donor

Parent-Child C+A reaction: K_2 = large, $k_2 =$ 2 10^4 $M^{-1}s^{-1}$

C homolytic cleavage: K_{hom} = large, $k_{hom} <$ 0.1 s^{-1} without proton donor and adjusted in the presence of proton donor

F/G electron transfer: E_3^0 = 0.315 V, $k_S = 10^4$ cm/s (Nernstian)

Homogeneous electron transfer F+B: equilibrium constant fixed by E^0 and $k_{dif} = 10^{10}$ $M^{-1}s^{-1}$

"DET" mechanism

$$\mathrm{A} + \mathrm{e} \xrightleftharpoons{E^0} \mathrm{B}$$

$$\mathrm{B} \xrightarrow{k_1(\mathrm{AH})} \mathrm{C}$$

$$\mathrm{C} + \mathrm{A} \xrightarrow{k_2} \mathrm{D}$$

$$\mathrm{C} + \mathrm{e} \xrightarrow[k_{\mathrm{S}}(\mathrm{AH})]{E^0{}_{\mathrm{DET}} >> E^0} \mathrm{G} + \text{leaving group}$$

A/B electron transfer: E^0 = -2.675 V, $k_S = 10^4$ cm/s (Nernstian)

B/C reaction: K_1 = large (no effect as soon as $K_1 > 10$), $k_1 =$ 160 s^{-1} without proton donor and adjusted in the presence of proton donor

Parent-Child C+A reaction: K_2 = large, $k_2 =$ 2 10^4 $M^{-1}s^{-1}$

C concerted reductive cleavage: E^0_{DET} = 1.05 V, MHL rate law, λ_{DET} = 4 eV and $k_{S,DET} < 10^{-21}$ cm/s in absence of proton donor and adjusted in the presence of proton donor.

6.2. PNO 1.15 mM no added water

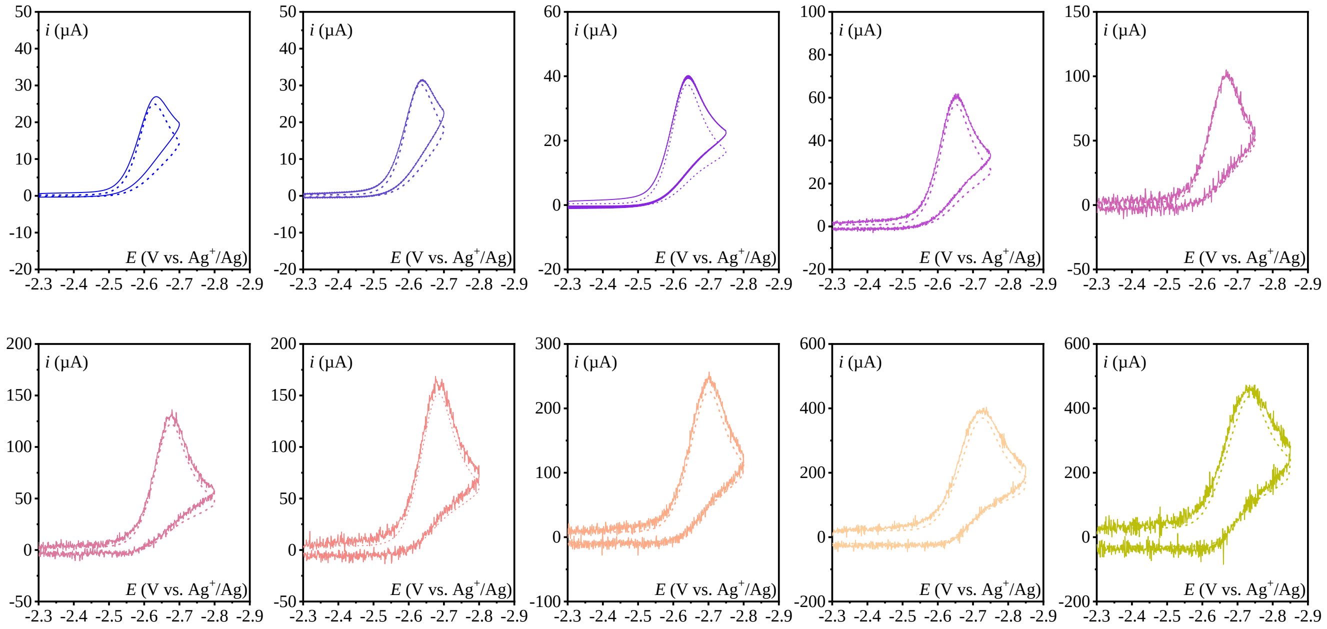

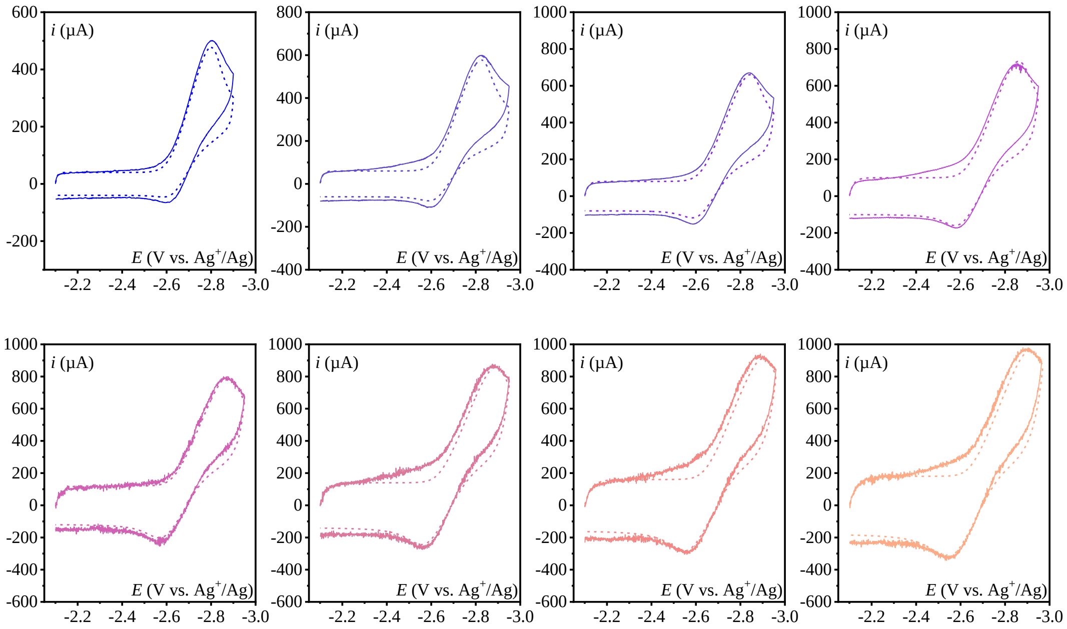

Fig. S7. CVs of PNO 1.15 mM in CH_3CN + 0.1 M *n*-Bu_4NPF_6 under Ar on a 3 mm diameter GCE at various scan rates. From top to bottom and left to right, ν = 0.05, 0.07, 0.1; 0.2, 0.5, 0.7, 1, 2, 5, 7, 10, 15, 20, 25, 30, 35, 40, 45 V/s. Dashed lines: simulations. Ohmic drop correction: from 0.05 to 7 V/s: 100 Ω ; above 7 V/s, no ohmic drop correction.

6.3. PNO 1.15 mM addition of water

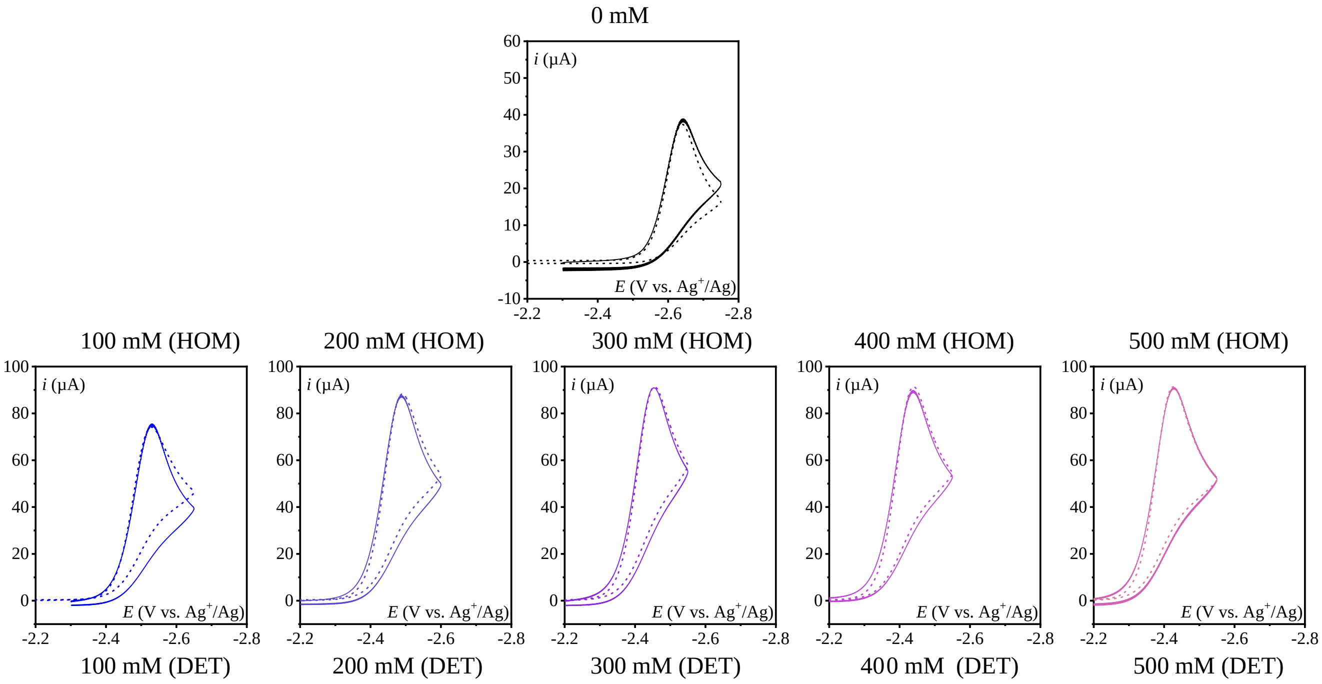

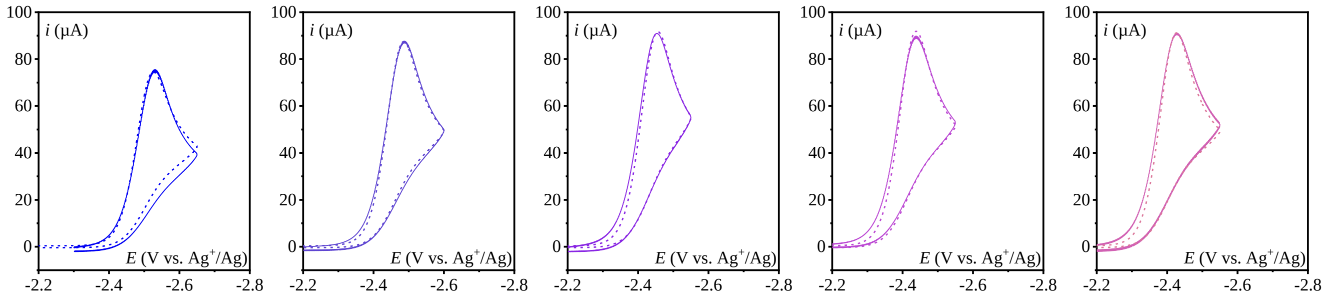

Fig. S8. CVs of PNO 1.15 mM in CH_3CN + 0.1 M *n*-Bu_4NPF_6 under Ar on a 3 mm diameter GCE at 0.1 V/s with addition of H_2O (from 0 to 500 mM). Dashed lines: simulations either considering HOM: homolytic cleavage of $PNOH^{\bullet}$ followed by reduction of $OH^{\bullet}$ or considering DET: concerted dissociative electron transfer for $PNOH^{\bullet}$.

6.4. PNO 1 mM no added water

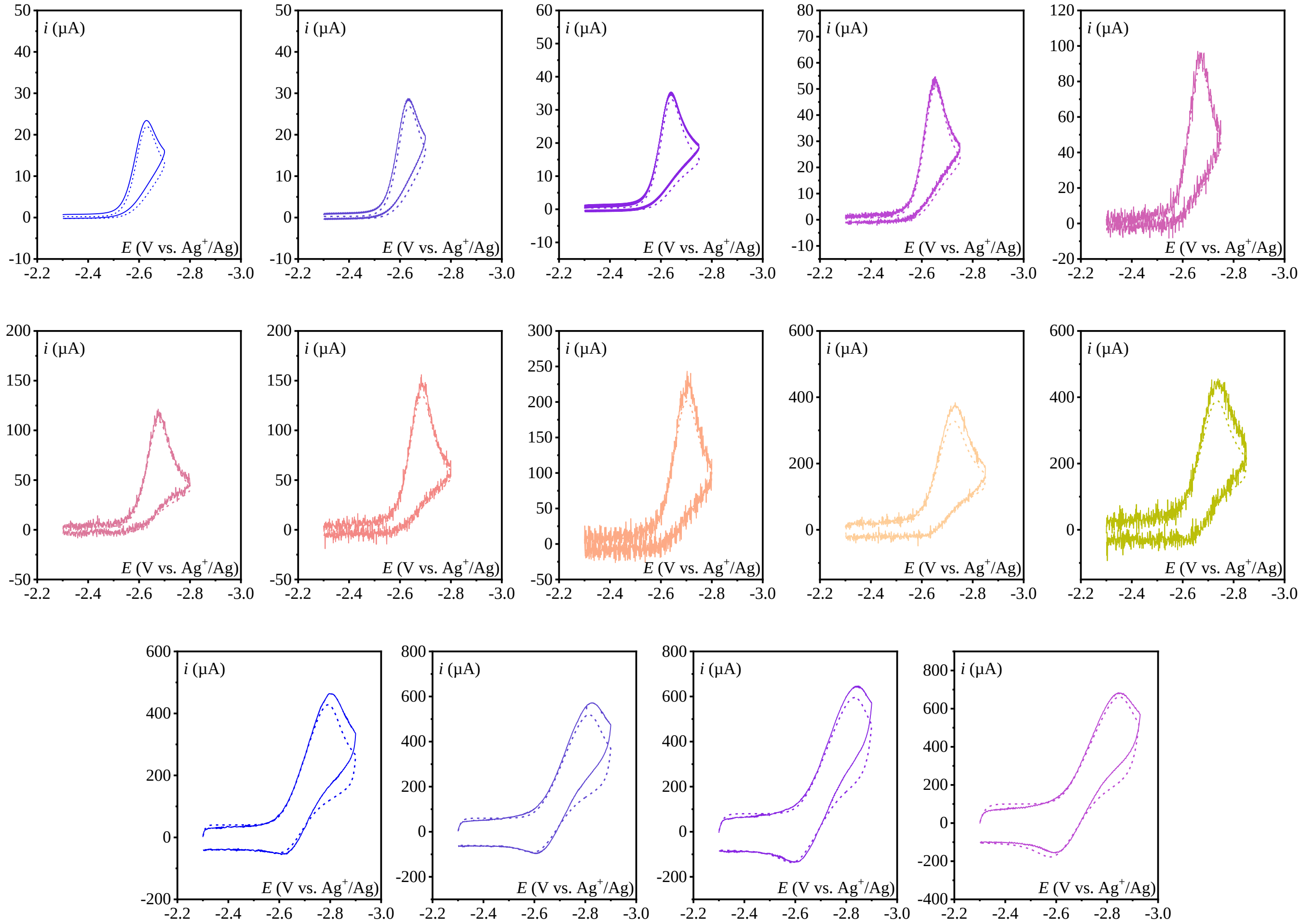

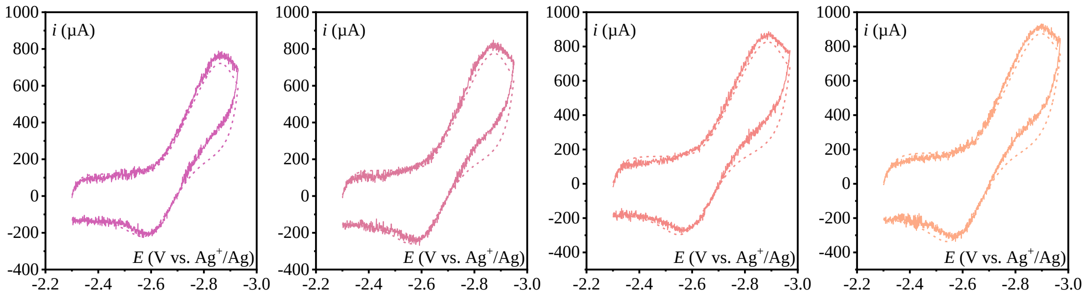

Fig. S9. CVs of PNO 1 mM in CH_3CN + 0.1 M *n*-Bu_4NPF_6 under Ar on a 3 mm diameter GCE at various scan rates. From top to bottom and left to right, ν = 0.05, 0.07, 0.1; 0.2, 0.5, 0.7, 1, 2, 5, 7, 10, 15, 20, 25, 30, 35, 40, 45 V/s. Dashed lines: simulations. Ohmic drop correction: from 0.05 to 7 V/s: 100 Ω; above 7 V/s, no ohmic drop correction.

6.5. PNO 1 mM addition of D_2O

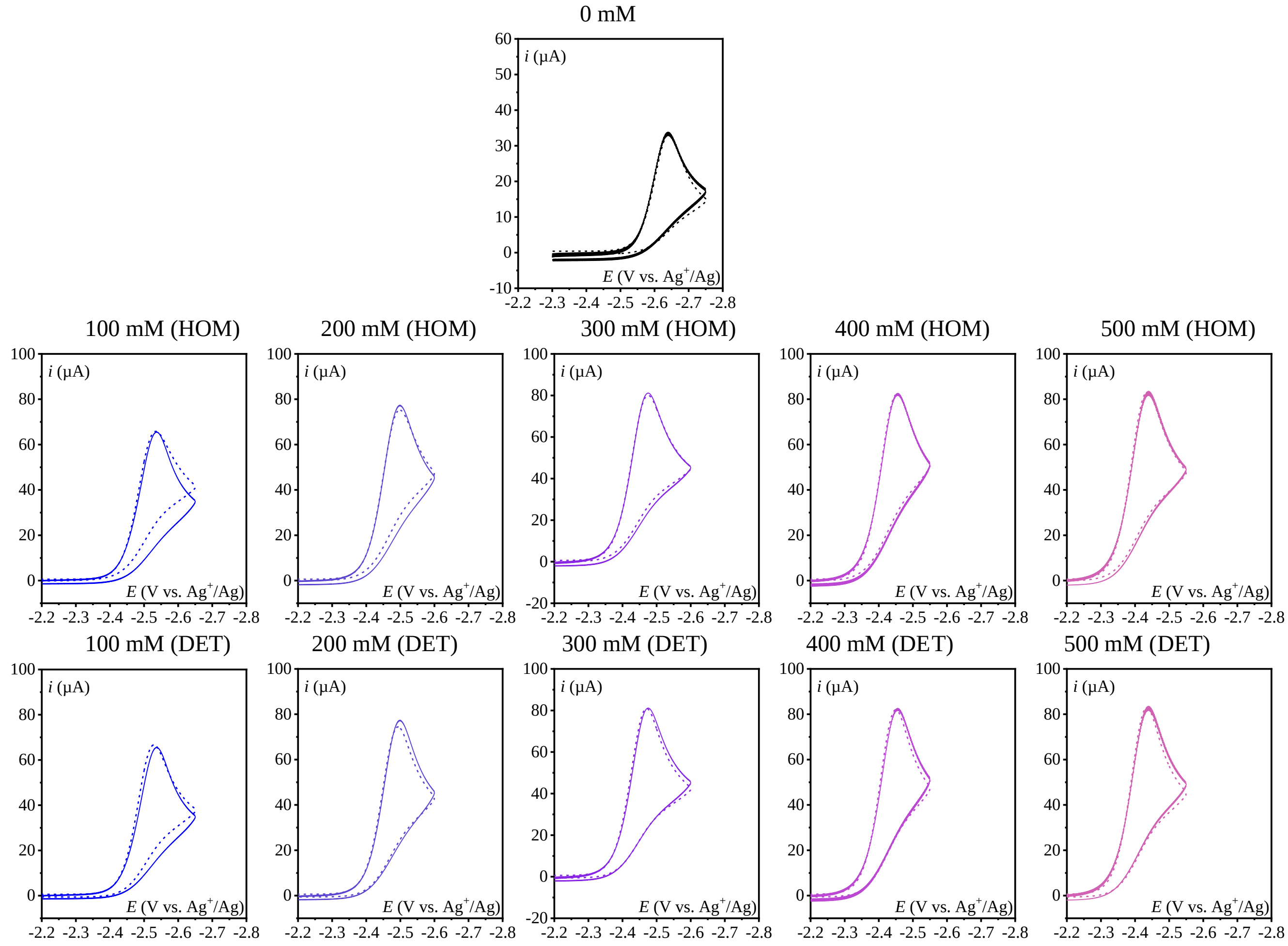

Fig. S10. CVs of PNO 1 mM in CH_3CN + 0.1 M *n*-Bu_4NPF_6 under Ar on a 3 mm diameter GCE at 0.1 V/s with addition of D_2O (from 0 to 500 mM). Dashed lines: simulations either considering HOM: homolytic cleavage of $PNOH^{\bullet}$ followed by reduction of $OH^{\bullet}$ or considering DET: concerted dissociative electron transfer for $PNOH^{\bullet}$.

6.6. PNO 1 mM addition of EtOH

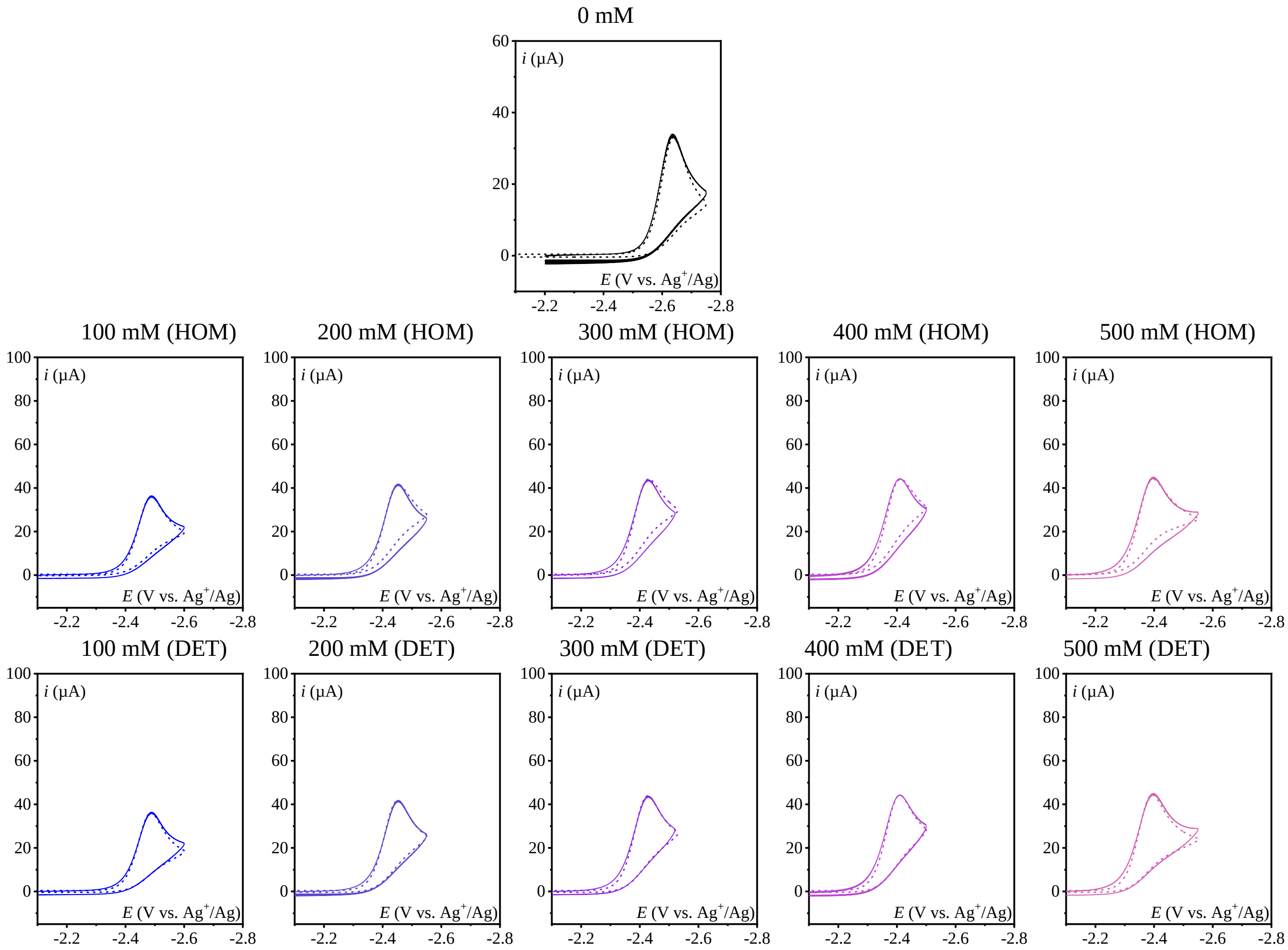

Fig. S11. CVs of PNO 1 mM in CH_3CN + 0.1 M *n*-Bu_4NPF_6 under Ar on a 3 mm diameter GCE at 0.1 V/s with addition of EtOH (from 0 to 500 mM). Dashed lines: simulations either considering HOM: homolytic cleavage of $PNOH^{\bullet}$ followed by reduction of $OH^{\bullet}$ or considering DET: concerted dissociative electron transfer for $PNOH^{\bullet}$.

7. Constant potential electrolysis without added acid

A controlled potential electrolysis at -2.72 V vs. Ag^+/Ag was run on a carbon felt in a 10 mL 5.75 mM PNO solution in CH_3CN + 0.1 M Bu_4NPF_6 under argon. The solution was stirred at 900 rpm.
The current drops to the background current (ca. 1 mA) after the passage of 11 C (after background charge correction) corresponding to 2 electrons per PNO (Figure S12). CVs run on a 3 mm GCE show the complete consumption of PNO after electrolysis.

According to the NMR titration before and after electrolysis a conversion yield was calculated as being 80%. Accordingly, due to the background current, the faradaic yield after 40 min electrolysis is 63.5 %.

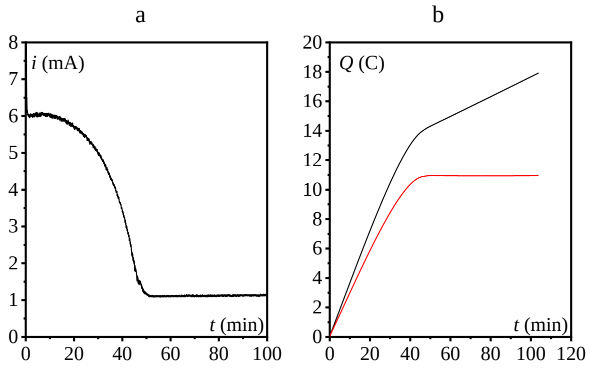

Fig. S12. (a) Current vs. time. (b) Charge vs. time. Raw data in black. Corrected charge in red obtained after subtraction of a background charge due to the background current on the carbon felt corresponding to 1 mA.

A controlled potential electrolysis at -2.63 V vs. Ag^+/Ag was run on a carbon felt in a 10 mL 10 mM PNO solution in CH_3CN + 0.1 M Bu_4NPF_6 under Ar. The solution was initially stirred at 900 rpm and then the stirring rate was modified every ca. 10 min thus being successively: 900, 200, 600, 300, 900, 500, 150, 900, 600, 900 rpm (Figure S13). It is seen that the effect of the rotation rate is minor in the first part of the electrolysis (when the current is slowly decreasing over time). The rotation rate as a major effect in the decreasing part of the current indicating that the current is then controlled by mass transport.

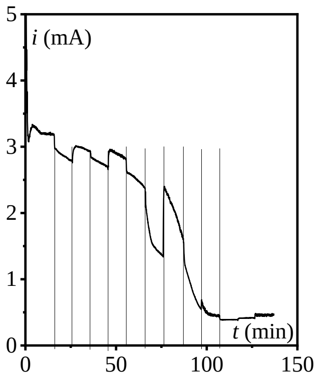

Fig. S13. Current vs. time with variable stirring rate of the solution.

8. CVs of PNO and Py in the presence of water

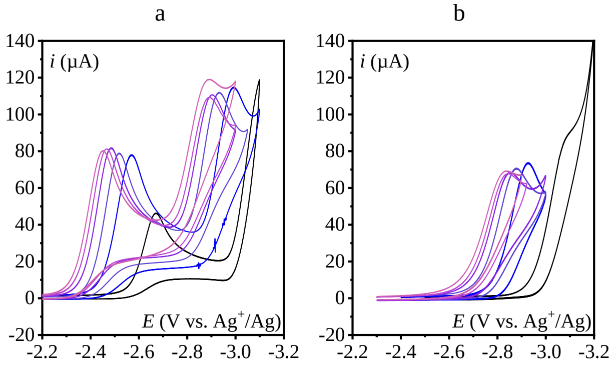

Fig. S14. CVs at 0.1 V/s on a 3 mm diameter GCE in CH_3CN + 0.1 M *n*-Bu_4NPF_6 under Ar. (a) PNO 1.2 mM with 0 (black), 100, 200, 300, 400, 500 mM of H_2O. (b) Py 1 mM with 0 (black), 100, 200, 300, 400, 500 mM of H_2O.

9. Constant potential electrolysis with added water

An exhaustive CPE was performed at -2.60 V vs. Ag^{+}/Ag on a carbon felt electrode using a 9.15 mM solution of PNO with 200 mM H_2O. According to the NMR titration before and after electrolysis a conversion yield was calculated as being 75 %. Accordingly, due to the background current, the faradaic yield after 100 min electrolysis is 55 %. CV after electrolysis shows the complete consumption of PNO. The oxidation wave at -0.36 V vs. Ag^{+}/Ag was also observed if electrolysis is run in the absence of PNO and results from reduction of the electrolyte on the carbon felt at this negative potential.

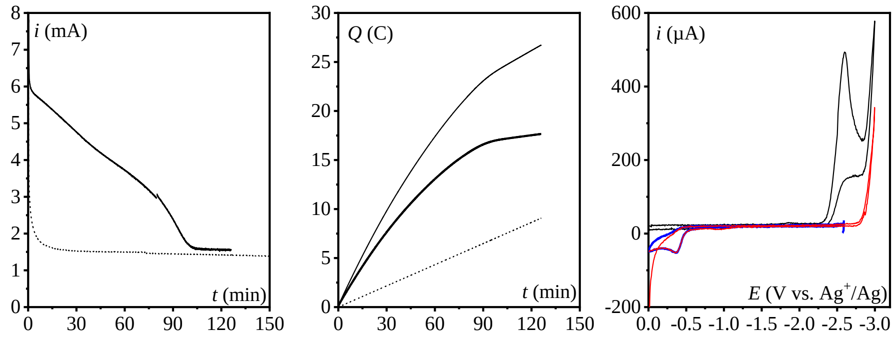

Fig. S15. (a) Current vs. time. Full line with PNO and added water. Dashed line: background current on the carbon felt without PNO. (b) Charge vs. time. Thin full line: with PNO and added water. Dashed line: background charge on the carbon felt without PNO. Thick full line: Corrected charge obtained after subtraction of a background charge. (c) CVs before (black) and after (red and blue) electrolysis recorded in the electrolysis cell with a 3 mm GCE. Initial potential and direction of initial scan: black: 0 V, cathodic direction; blue: -2.6 V, anodic direction; red: 0 V, cathodic direction.

10. DFT calculations

10. 1 General consideration

Calculations were performed using the ORCA 5.0 suite of software. [1S] The M06 functional [2S] with Grimme's D3 dispersion correction [3S] was used in conjunction with the 6-311+G(2df,2p) basis set for all atoms. [4S] All geometries were fully optimized without any symmetry or geometry constraints. Harmonic vibrational analyses were performed to confirm and characterize the structures as minima. Free energies were calculated within the harmonic approximation for vibrational frequencies. The effects of the solvation by acetonitrile were included in the energy calculations using the CPCM model. [5S] Standard potentials were calculated with respect to the phenazine$^{0/–}$ redox couple (E^0= –1.611 V vs. Fc^+/Fc) and converted back versus the $Fc^{+/0}$ redox couple as previously described for accuracy (converted in the manuscript to Ag^+/Ag). [6S] The calculation parameters were selected for their good performance in describing anionic organic species. [7S]

Scheme S5

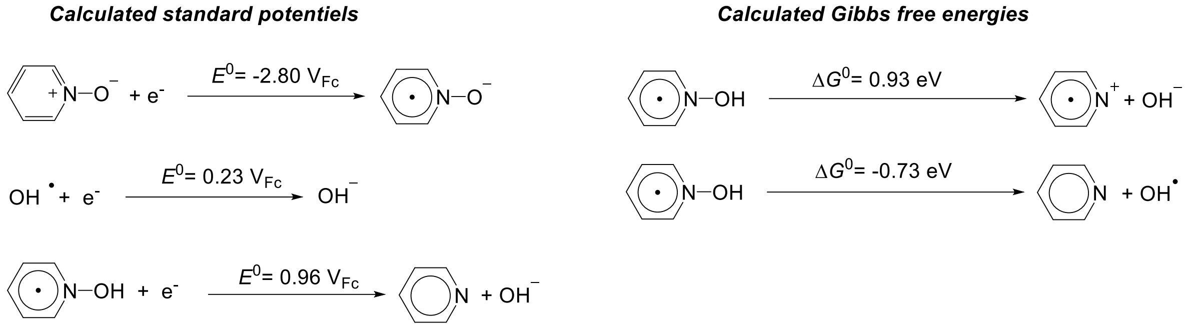

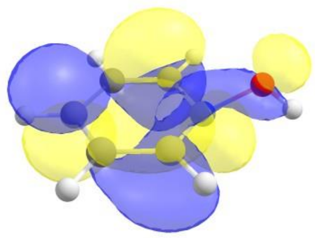

Fig. S16. Computed electron density of the SOMO of PNOH$^{\bullet}$ showing the out-of-the-plane configuration and the contribution to the σ* N–O bond.

10.2 Three lowest frequencies and Gibbs free energy for all computed structures

Structure geometries can be found in the xyz document .

PNO

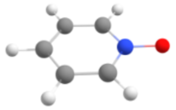

```
225.75 cm**-1
415.21 cm**-1
478.01 cm**-1
Final Gibbs free energy         ...    -323.28015035 Eh
```

PNO$^{\bullet -}$

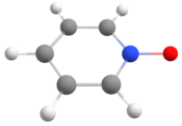

```
103.28 cm**-1
178.39 cm**-1
425.43 cm**-1
Final Gibbs free energy         ...    -323.35903894 Eh
```

PNOH$^{\bullet}$

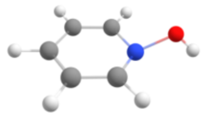

```
112.57 cm**-1
245.89 cm**-1
337.88 cm**-1
Final Gibbs free energy          ...   -323.82799755 Eh
```

Py

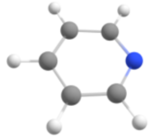

```
379.65 cm**-1
420.15 cm**-1
602.15 cm**-1
Final Gibbs free energy          ...   -248.11557952 Eh
```

Py$^{•+}$

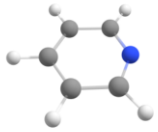

```
370.04 cm**-1
408.65 cm**-1
581.94 cm**-1
Final Gibbs free energy          ...   -247.86429196 Eh
```

Phenazine

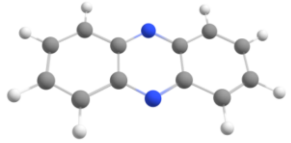

```
92.60 cm**-1
106.28 cm**-1
236.76 cm**-1
Final Gibbs free energy          ...   -571.22023408 Eh
```

Phenazine$^{•-}$

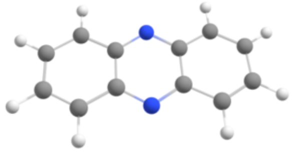

```
91.41 cm**-1
102.74 cm**-1
243.47 cm**-1
Final Gibbs free energy          ...   -571.34270346 Eh
```

HO$^{•}$

```
3739.86 cm**-1
Final Gibbs free energy          ...    -75.73934120 Eh
```

HO^{-}

```
3925.99 cm**-1
Final Gibbs free energy          ...    -75.92948781 Eh
```

11. References

1S. Neese, F., Software update: The ORCA program system—Version 5.0. *WIREs Computational Molecular Science* **2022**, *12*, e1606

2S. Zhao Y.; Truhlar, D. G., The M06 suite of density functionals for main group thermochemistry, thermochemical kinetics, noncovalent interactions, excited states, and transition elements: two new functionals and systematic testing of four M06-class functionals and 12 other functionals. *Theor. Chem. Acc.* **2008**, *120*, 215-241.

3S. Grimme, S.; Antony, J.; Ehrlich, S.; Krieg, H. A Consistent and Accurate Ab Initio Parametrization of Density Functional Dispersion Correction (DFT-D) for the 94 Elements H-Pu. *J. Chem. Phys.* **2010**, *132*, 154104.

4S. (a) Krishnan, R.; Binkley, J. S.; Seeger, R.; Pople, J. A., Self-consistent molecular orbital methods. XX. A basis set for correlated wave functions. *J. Chem. Phys.* **1980**, *72*, 650-654. (b) McLean, A. D.; Chandler, G. S. Contracted Gaussian Basis Sets for Molecular Calculations. I. Second Row Atoms, Z=11–18. *J. Chem. Phys.* **1980**, *72*, 5639–5648. (c) Curtiss, L. A.; McGrath, M. P.; Blaudeau, J. P.; Davis, N. E.; Binning, R. C.; Radom, L., Extension of Gaussian-2 theory to molecules containing third-row atoms Ga–Kr. *J. Chem. Phys.* **1995**, *103*, 6104-6113. (d) Clark, T.; Chandrasekhar, J.; Spitznagel, G. W.; Schleyer, P. V. R., Efficient diffuse function-augmented basis sets for anion calculations. III. The 3-21+G basis set for first-row elements, Li–F. *J. Comput. Chem.* **1983**, *4*, 294-301. (e) Frisch, M. J.; Pople, J. A.; Binkley, J. S., Self-consistent molecular orbital methods 25. Supplementary functions for Gaussian basis sets. *J. Chem. Phys.* **1984**, *80*, 3265-3269. (f) Weigend, F., Accurate Coulomb-fitting basis sets for H to Rn. *Phys. Chem. Chem. Phys.* **2006**, *8*, 1057-1065.

5S. Cossi, M.; Rega, N.; Scalmani, G.; Barone, V., Energies, structures, and electronic properties of molecules in solution with the C-PCM solvation model. *J. Comput. Chem.* **2003**, *24* , 669-681.

6S. Moreno, J. J.; Hooe, S. L.; Machan, C. W., DFT Study on the Electrocatalytic Reduction of CO_2 to CO by a Molecular Chromium Complex. *Inorg. Chem.* **2021**, *60*, 3635-3650.

7S. Borioni, J. L.; Puiatti, M.; Vera, D. M. A.; Pierini, A. B. In search of the best DFT functional for dealing with organic anionic species *Phys. Chem. Chem. Phys.* 2017, *19*, 9189–9198.
